# Supplementary material for: Control of tissue flows and embryo geometry in avian gastrulation
Source: Nat Commun. 2025 Jun 4;16:5174. doi: 10.1038/s41467-025-60249-8 (PMC12137940; doi:10.1038/s41467-025-60249-8)
Supplement: Supplementary file 1 — Supplementary Information [file 41467_2025_60249_MOESM1_ESM.pdf]

Supplementary Information for
Control of Tissue Flows and Embryo Geometry in Avian Gastrulation

Guillermo Serrano Nájera*[1†], Alex M. Plum*[2], Ben Steventon[1], Cornelis J. Weijer[3†], Mattia Serra[2†]

[1]Department of Genetics, University of Cambridge, Cambridge CB2 3EH, UK
[2]Department of Physics, University of California San Diego, CA 92093, USA
[3]Division of Molec. Cell and Dev. Biology, School of Life Sciences, Univ. of Dundee, UK
*Equal Contribution.
†Emails for correspondence: gs714@cam.ac.uk, c.j.weijer@dundee.ac.uk, mserra@ucsd.edu

Contents

S1 The Dynamic Morphoskeleton **1**
S1.1 DM and Eulerian quantities 2

S2 Mathematical Model **4**
S2.1 Active Stress Magnitude 4
S2.2 Active Stress Orientation and Anisotropy 5
S2.3 Force Balance 7
S2.4 Analysis in 1D and Biophysical Constraints on α and β 7
S2.5 Dimensionless 2D Model 8
S2.6 Boundary Conditions 8
S2.7 Initial Conditions 8
S2.8 Numerical Scheme 9

S3 Model Perturbations **10**
S3.1 Active Forces 11
S3.2 Connection Between R2 and the Attractor 11

S4 Note on Epiboly and Evolution **12**

S5 Experimental Methods **13**
S5.1 *Ex Ovo* Culture 14
S5.2 Mechanical Confinement and Chemical Perturbations 14
S5.3 Velocimetry 14
S5.4 Determination of Embryo Proper, and Extraembryonic Areas Over Time 14
S5.5 Immunohistochemistry 14
S5.6 Computational Surface Extraction 14
S5.7 Statistics and Reproducibility 15

S6 Additional Supplementary Figures & Tables **16**

S1 The Dynamic Morphoskeleton

The Dynamic Morphoskeleton (DM) locates dynamic attractors and repellers from tissue velocities [1] or cell trajectories [2]. Using only kinematic data, the DM is agnostic to the forces and microscopic mechanisms underlying coherent

cell motion and can be computed over any developmental interval $[t_0, t]$ in the dataset. We denote the cell trajectories by

$$\mathbf{F}_{t_0}^t(\mathbf{x}_0) = \mathbf{x}_0 + \int_{t_0}^{t} \mathbf{v}\big(\mathbf{F}_{t_0}^{\tau}(\mathbf{x}_0), \tau\big)\mathrm{d}\tau, \tag{S1}$$

marking the time$-t$ positions of cells that started at $\mathbf{x}_0$ at initial time t_0 (Fig. S1A). The largest singular value ${}_2\lambda_{t_0}^t(\mathbf{x}_0)$ of the deformation gradient $\boldsymbol{\nabla}_{\mathbf{x}_0}\mathbf{F}_{t_0}^t(\mathbf{x}_0)$ quantifies the maximum separation by time t between neighboring trajectories starting near $\mathbf{x}_0$ at t_0

$${}_2\lambda_{t_0}^t(\mathbf{x}_0) = \max_{\delta\mathbf{x}_0} \frac{\overbrace{|\boldsymbol{\nabla}_{\mathbf{x}_0}\mathbf{F}_{t_0}^t(\mathbf{x}_0)\delta\mathbf{x}_0|}^{\delta\mathbf{x}_t}}{|\delta\mathbf{x}_0|}, \tag{S2}$$

where $\delta\mathbf{x}_0$ parameterizes the neighborhood of $\mathbf{x}_0$. Repellers are identified from the ridges (i.e. regions of high values) of ${}_2\lambda_{t_0}^t(\mathbf{x}_0)$, following trajectories forward in time (Fig. S1B). Likewise, attractors are computed from the flow map's inverse, $\mathbf{F}_t^{t_0}(\mathbf{x}_t)$, tracing trajectories back in time, and located by ridges of ${}_2\lambda_t^{t_0}(\mathbf{x}_t)$ denoting the largest singular value of $\boldsymbol{\nabla}_{\mathbf{x}_t}\mathbf{F}_t^{t_0}(\mathbf{x}_t)$. For more details and algorithm, see [1]. ${}_2\lambda_{t_0}^t(\mathbf{x}_0)$ is related to the finite time Lyapunov exponent (FTLE) by $\mathrm{FTLE}(\mathbf{x}_0) = \log {}_2\lambda_{t_0}^t(\mathbf{x}_0)/|t - t_0|$. Taking the logarithm is appropriate in chaotic flows characterized by exponential stretching, but for slow morphogenetic flows, ${}_2\lambda_{t_0}^t(\mathbf{x}_0)$ is more appropriate. Moreover, because we are interested in the presence or absence of the repellers and their average geometry, in the main text, we normalize ${}_2\lambda_{t_0}^t(\mathbf{x}_0)$ in each plot by its maximum spatial value. See Fig. S11 for absolute levels, exhibiting similar magnitudes of cumulative deformation in model and experiments. Fitting the experimental values precisely is less informative as the approximation of ${}_2\lambda_{t_0}^t(\mathbf{x}_0)$ from experimental velocity data can be affected by its resolution [3], whereas the average geometry remains robust (Fig. S8).

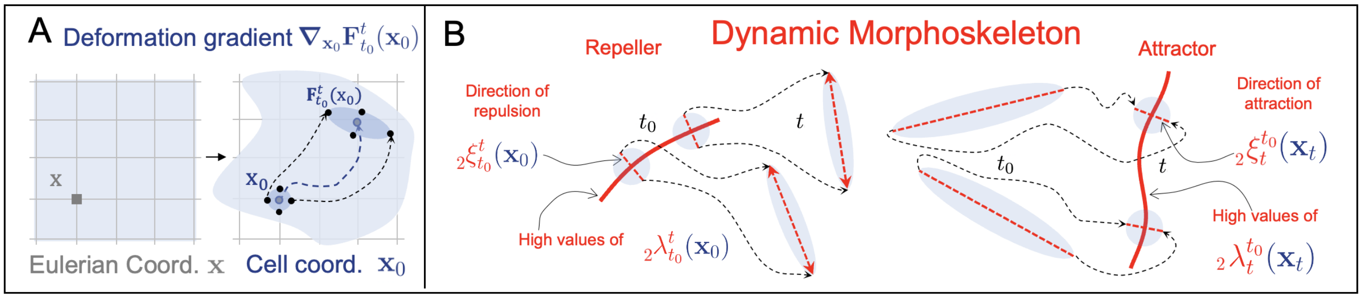

Supplementary Figure S1: **The Dynamic Morphoskeletons [1] Quantify Morphogenetic Flows.** A) Eulerian coordinates $\mathbf{x}$ describe fixed spatial locations, while Lagrangian (cell) coordinates $\mathbf{x}_0$ label the identity of cells or tissue regions at their initial position and follow their trajectories $\mathbf{F}_{t_0}^t(\mathbf{x}_0)$. B) Fix the initial time t_0 of the Lagrangian analysis, which we consider to be the beginning of our experiments (edevelopmental stage HH1). For any Lagrangian timescale $t - t_0$, the Dynamic Morphoskeleton consists of repellers and attractors. Repellers mark regions at the initial cell configuration $\mathbf{x}_0$ across which cells maximally separate by time t. Attractors mark regions on the final tissue configuration towards which cells maximally converge by time t.

The DM *i)* is invariant to time-dependent translations and rotations of the (arbitrary) coordinate system used to describe cell motion and global artifacts such as embryo drifts, enabling robust comparative analyses, which would otherwise require careful spatiotemporal alignment of the embryos at each time point; *ii)* is integrative, providing aggregate information along trajectories, guaranteeing robust results even with low-resolution wide-field microscopy; *iii)* reduces spatiotemporal velocities into discrete, interpretable units. We illustrate these concepts below.

S1.1 DM and Eulerian quantities

We use chick gastrulation velocities (Fig. S2, [1]) to facilitate connections and differences between the DM and Eulerian quantities. *1)* Velocity plots are unsuitable for locating regions of convergence and separation in time-dependent flows. For example, at the primitive streak (inside the red rectangle in Fig. S2A), the tissue is highly converging without revealing a clear signature in $\mathbf{v}$. *2)* Instead, one should look at the frame invariant rate-of-strain tensor $\mathbf{S}(\mathbf{x}, t) = 1/2(\nabla\mathbf{v}(\mathbf{x}, t) + [\nabla\mathbf{v}(\mathbf{x}, t)]^\top)$, (Fig. S2B-C) to locate local (in space and time) attraction or repulsion regions, as recently found [4, 5]. Specifically, denoting by $s_1(\mathbf{x}, t) \leq s_2(\mathbf{x}, t)$ the eigenvalues of $\mathbf{S}(\mathbf{x}, t)$, regions of low $s_1(\mathbf{x}, t)$

(high $s_2(\mathbf{x},t)$) mark short time attractors (repellers), and the associated eigenvectors of $\mathbf{S}(\mathbf{x},t)$ indicate the direction of convergence (separation). Indeed, Fig. S2B shows high local convergence (or a short-time attractor [6]) at the Prim-

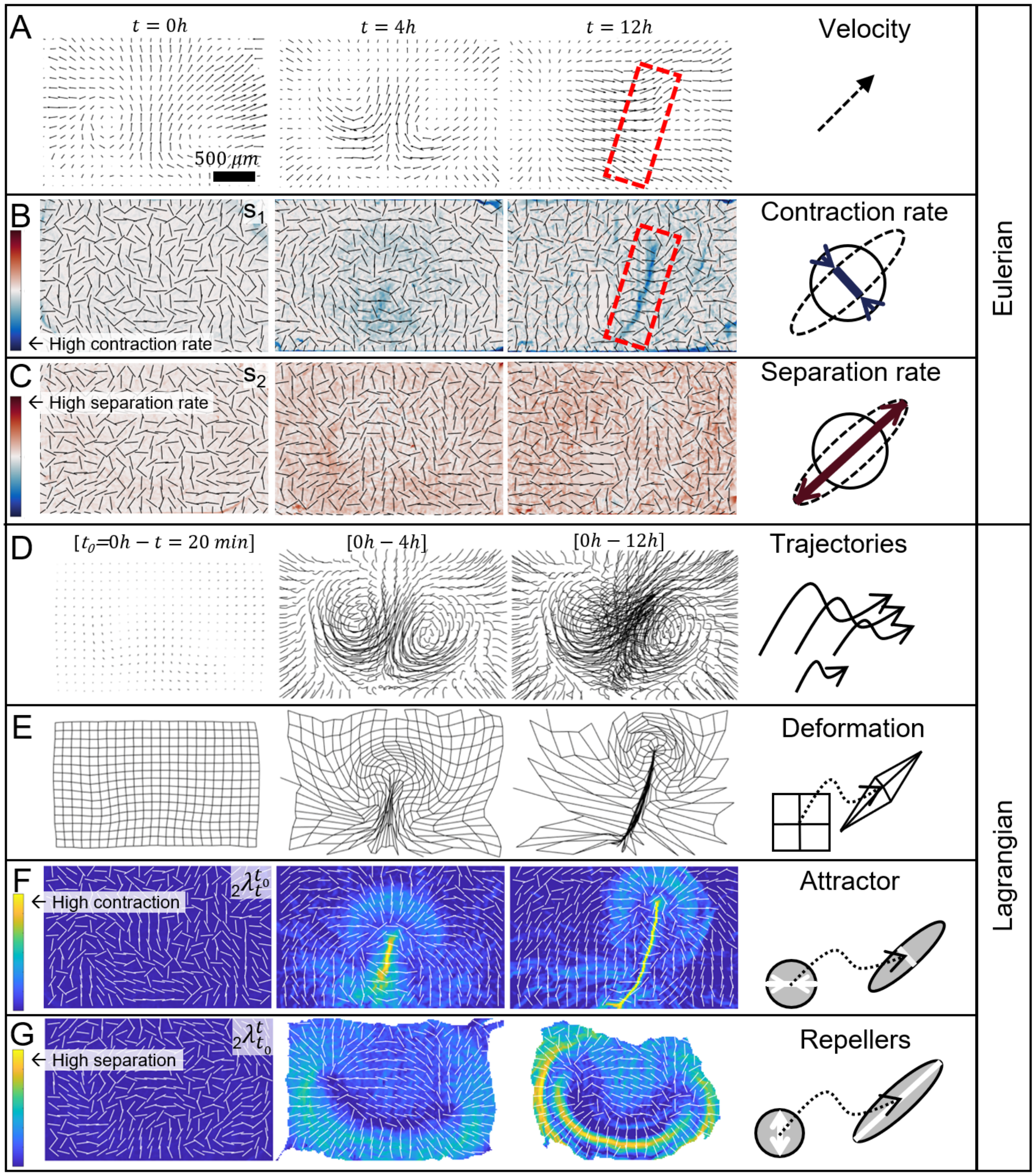

Supplementary Figure S2: **Connections between DM and Eulerian quantities in Chick Gastrulation.** A) Chick Gastrulation Experimental velocities $\mathbf{v}(\mathbf{x},t)$. t_0 corresponds to developmental stage HH1, and the red rectangle contains the Primitive Streak. B) $s_1(\mathbf{x},t)$ field and directions (white bars) show the smallest eigenvalues (eigenvector) of the rate of strain tensor $\mathbf{S}(\mathbf{x},t) = 1/2(\nabla\mathbf{v}(\mathbf{x},t) + [\nabla\mathbf{v}(\mathbf{x},t)]^\top)$, showing the direction and intensity of maximal contraction rates. C) Same as B for maximal stretching rates and directions. D) Cell Trajectories. E) Deforming Lagrangian grid. F) Largest contraction ratio ${}_2\lambda_t^{t_0}(\mathbf{x}_t)$ along cell trajectories (Eq. (S2)) and contracting direction (white bar). High values of ${}_2\lambda_t^{t_0}(\mathbf{x}_t)$ mark Attractors. G) Largest separation ratio ${}_2\lambda_{t_0}^t(\mathbf{x}_0)$ along cell trajectories and expanding direction (white bar). High values of ${}_2\lambda_{t_0}^t(\mathbf{x}_0)$ mark Repellers.

itive Streak, where $\mathbf{v} \neq \mathbf{0}$ (Fig. S2A). This observation reveals that regions in deforming tissues can undergo large

attraction/repulsion while they move. By contrast, standard methods incorrectly look at frame-dependent saddle-type fixed points of the velocity (i.e., where $\mathbf{v} = \mathbf{0}$) to locate regions of the flows that undergo large convergence/separation, leading to incorrect predictions as recently recognized [7]. *3)* The (Eulerian) rate of strain tensor is frame invariant yet agnostic to cell paths. Tissue patches, instead, move and integrate deformations along their trajectories (Fig. S2D) as visualized by a deforming Lagrangian grid (Fig. S2E). The DM, made of attractors and repellers and computed from Eq. (S2), precisely accounts for this cumulative deformation, revealing the intensity and direction of attraction and repulsion of tissue patches over a finite time (Fig. S2E-G). Comparing Fig. S2E-G with Fig. S2B-C shows how the DM reveals robust morphogenetic features that arise and shape over time, not contained in Eulerian quantities.

Kinematics vs Forces. Just as knowledge and inspection of Eulerian velocities do not reveal the underlying forces generating them (e.g. if cells moving with locally observed velocities are actively pulling or passively pushed), knowledge of attractors and repellers does not translate to knowledge of the underlying forces causing them or the presence of colocated active forces pulling towards and pushing away nearby tissues. Summarizing spatiotemporal velocities with three kinematic units (2 repellers and one attractor), however, guided our experiment design and force-based model development, enabling us to uncover their generating mechanisms and independent controllability.

Limitations of the DM. Contrary to Eulerain methods, quickly computable from snapshots of $\mathbf{v}$ and straightforward to visualize, the DM and Lagrangian methods are more computationally demanding, requiring trajectory calculations. Additionally, owing to the finite-time integrative nature of the DM, care must be taken when choosing an appropriate time interval. If the time interval is too short, it may fail to resolve Lagrangian features that develop over time. If it is too long, it may fail to capture transient changes of $\mathbf{v}$ over time. In general, there is a many-to-one relationship between spatiotemporal velocity fields and cumulative deformation, limiting straightforward kinematic interpretation of the DM. This can be addressed by studying how the DM develops over time and combining it with Eulerian kinematic analyses. When the goal is detecting temporal changes in tissue flows [8] and velocity spatial patterns [9, 10], rather than their induced cumulative deformations, Eulerian snapshots of $\mathbf{v}$, streamlines, vorticity or short-time attractors and repellers [4, 5] are more appropriate. In addition to the above limitations, computing the DM further demands taking spatial derivatives of trajectory maps with respect to initial conditions (Eq. S2), which requires dedicated methods when trajectories are noisy and sparse [2, 3]. Last, automatic extraction of ridges (as parametrized curve or surfaces) from scalar fields—including attractors and repellers—remains a challenging task [11].

S2 Mathematical Model

Avian gastrulation flows (planar velocity $\mathbf{v}(\mathbf{x}, t) = [u(\mathbf{x}, t), v(\mathbf{x}, t)]$) are driven by external boundary conditions (epiboly motion) and forces arising from gradients of active stresses. We model active stresses arising from myosin activity in a compressible Stokes flow, as in our previous work [12]. In the following sections, we recap our model and extend it in two key ways: *i)* Expand our domain to account for both the embryo proper (EP) and the surrounding extraembryonic (EE) tissue and model their distinct dynamics. *ii)* Model the dynamic anisotropy of active stress in addition to its intensity and average orientation. These extensions are necessary to understand the mechanistic origins of Repeller 1 (R1), Repeller 2 (R2) and the embryo's dynamic geometry. See Table 4 for a map between old [12] and new parameters.

S2.1 Active Stress Magnitude

Active stresses are generated by non-muscle myosin II (hereafter just myosin) contracting F-actin bundles at adherens junctions in the cells' apical cortex [13, 14]. The amount of active myosin depends on *i)* the total myosin available for recruitment and activation and *ii)* the fraction of myosin activated. Since it is not well understood how *i)* changes during gastrulation, we assume that available myosin remains constant. Similarly, actin density is not explicitly modeled, as active myosin is assumed to be the limiting factor in active force generation. Thus, increases in myosin activity correspond to increases in the fraction activated. We define $m(\mathbf{x}, t)$ as the fraction of available myosin active in a patch of tissue. Our previous work defined m in units of active stress, introducing the overall scale of myosin activity as an additional variable, controlled by the maximum available myosin concentration m_n [12]. Here, by defining m as a fraction of maximum available myosin, we eliminate this degree of freedom and instead convert myosin activity into isotropic and anisotropic stress with two constant parameters α, β (units of stress) defined in Section S2.3. This retains the same physics and is equivalent to non-dimensionalizing m in [12] using m_n and re-scaling other parameters accordingly.

The active stress magnitude, proportional to $m(\mathbf{x}, t)$, evolves as

$$\partial_t m = \chi_1(1-m) - \chi_2 m e^{-\chi_3 m} - [\mathbf{v}\cdot\boldsymbol{\nabla}]m + \epsilon\Delta_{\mathbf{Q}} m. \tag{S3}$$

where χ_1 and χ_2 are effective rate parameters for the active stress dynamics due to activation and deactivation of myosin in a patch of tissue. Generating tissue-scale active stresses involves myosin binding to actin, phosphorylation, power strokes, and other mechanical processes [12,14,15]. The kinetics of these processes remain challenging to coarsen into tissue-scale models. In the chick embryo, successive, active intercalations can take up to an hour [14], consistent with other tissue-scale models in which myosin dynamics occur on the slowest timescale [16–18]. χ_{1-3} are chosen to ensure that *i)* the tissue-scale myosin dynamics preserve an active stress instability [12] (Fig. S3A) and *ii)* m does not saturate throughout the EP during gastrulation, consistent with experiments [12]. Total myosin availability could also exhibit heterogeneous dynamics associated with concurrent cell differentiation (i.e., mesendoderm cells may have higher availability). Yet, because such dynamics are not well characterized, we capture the key phenomenological features with Eq. (S3). m grows at a rate proportional to $1-m$, ceasing when all myosin is active ($m = 1$) and reaching its maximum rate when all myosin is inactive ($m = 0$). Our model requires an active stress instability to reproduce observed tissue flows and active stress patterns. Without this instability, gastrulation flows would cease (Table 3M). The instability arises from an unstable fixed point in the myosin kinetics (Fig. S3A), achieved in Eq. (S3) via negative feedback on detachment. Alternative kinetics, such as positive feedback on recruitment could also suffice, but negative feedback on detachment is supported by experiments. In particular, myosin's detachment rate exhibits mechanosensitivity, decreasing exponentially with tension [19], possibly due to a catch bond mechanism [20]. Because myosin activity largely determines junctional tension [21], the myosin deactivation rate decreases exponentially with m, with sensitivity χ_3 as shown in [12]. The first three terms of Eq. (S3) represent average cell-autonomous dynamics in the Lagrangian (or cell) frame. Examining their spatially uniform fixed points reveals an instability (m^*) between two stable equilibria [12]: a lower equilibrium corresponding to low baseline myosin activity and a higher equilibrium near saturation (≈ 1) (Fig. S3A). The last term accounts for the directed propagation of active stress intensity along actomyosin cables [12]. We define $\Delta_{\mathbf{Q}} m = \boldsymbol{\nabla}\cdot\big(\big[\mathbf{Q} + \frac{s}{2}\mathbf{I}\big]\boldsymbol{\nabla} m\big)$ to represent directional tension propagation proportional to the degree of nematic order (our proxy for the presence of cables, see Section S2.2). For example, if $\phi = 0$ (along x), $\Delta_{\mathbf{Q}} m = \boldsymbol{\nabla}\cdot([s\ 0;\ 0\ 0]\boldsymbol{\nabla} m)$, ensuring propagation only in x. ϵ is the ratio between tension-propagated myosin activity and transport via advection, which we take to be small, as explained in [12].

S2.2 Active Stress Orientation and Anisotropy

In addition to the amount of myosin activity on junctions, tissue stresses depend on its local distribution across junctions. Cortical actomyosin can rigidify an epithelium when isotropically distributed, raising the energy barrier for neighbor exchanges and reducing cell rearrangements [22, 23]. Conversely, anisotropic distributions can drive fluid-like motion, channeling cell rearrangements in certain directions to produce tissue-scale anisotropic flows [22,23]. The orientation and anisotropy of junctional actomyosin strongly affect the orientation and anisotropy of junctional tension [14, 21] and can sculpt cell shapes [24], orient divisions [25], and direct intercalations [14, 26, 27]. In the avian embryo, the most coherent motifs of myosin anisotropy are supracellular actomyosin cables spanning 2-8 cells, appearing perpendicular to the axis of the incipient primitive streak at the onset of mesoderm contraction [14]. Actomyosin cables are suggested to self-organize in a tension-dependent manner, leading to spontaneous large-scale anisotropies observed in avian gastrulation [12, 18, 28] and *Drosophila* germband extension [29–32]. To capture the self-organization of actomyosin cables, we treat actomyosin cables as elongated fibers and model their anisotropy (local nematic order) and orientation with a traceless, symmetric tensor $\mathbf{Q} = \frac{s}{2}[\cos 2\phi\ \ \sin 2\phi;\ \sin 2\phi\ \ -\cos 2\phi]$. Here, $\phi = \frac{1}{2}\arctan(Q_{11}/Q_{12})$ represents the average orientation of actomyosin cables and $s = 2\sqrt{Q_{11}^2 + Q_{12}^2}$ the degree of local cable alignment. Minimally, $\mathbf{Q}$ evolves due to flow coupling, active alignment, and passive relaxation:

$$\partial_t \mathbf{Q} = s\mathbf{D}_d + \mathbf{Q}\mathbf{W} - \mathbf{W}\mathbf{Q} + \big[\gamma m - \delta s^2\big]\mathbf{Q}, \tag{S4}$$

with vorticity tensor $\mathbf{W} = \frac{1}{2}(\boldsymbol{\nabla}\mathbf{v} - \boldsymbol{\nabla}\mathbf{v}^\top)$ and deviatoric strain rate tensor $\mathbf{D}_d = \frac{1}{2}(\boldsymbol{\nabla}\mathbf{v} + \boldsymbol{\nabla}\mathbf{v}^\top) - \frac{\boldsymbol{\nabla}\cdot\mathbf{v}}{2}\mathbf{I}$. The first term couples cable orientation and anisotropy to the shear rate $\mathbf{D}_d$. The second two terms account for rigid body rotation. The final term addresses nematic dynamics independent of tissue flows and only affects s, as becomes

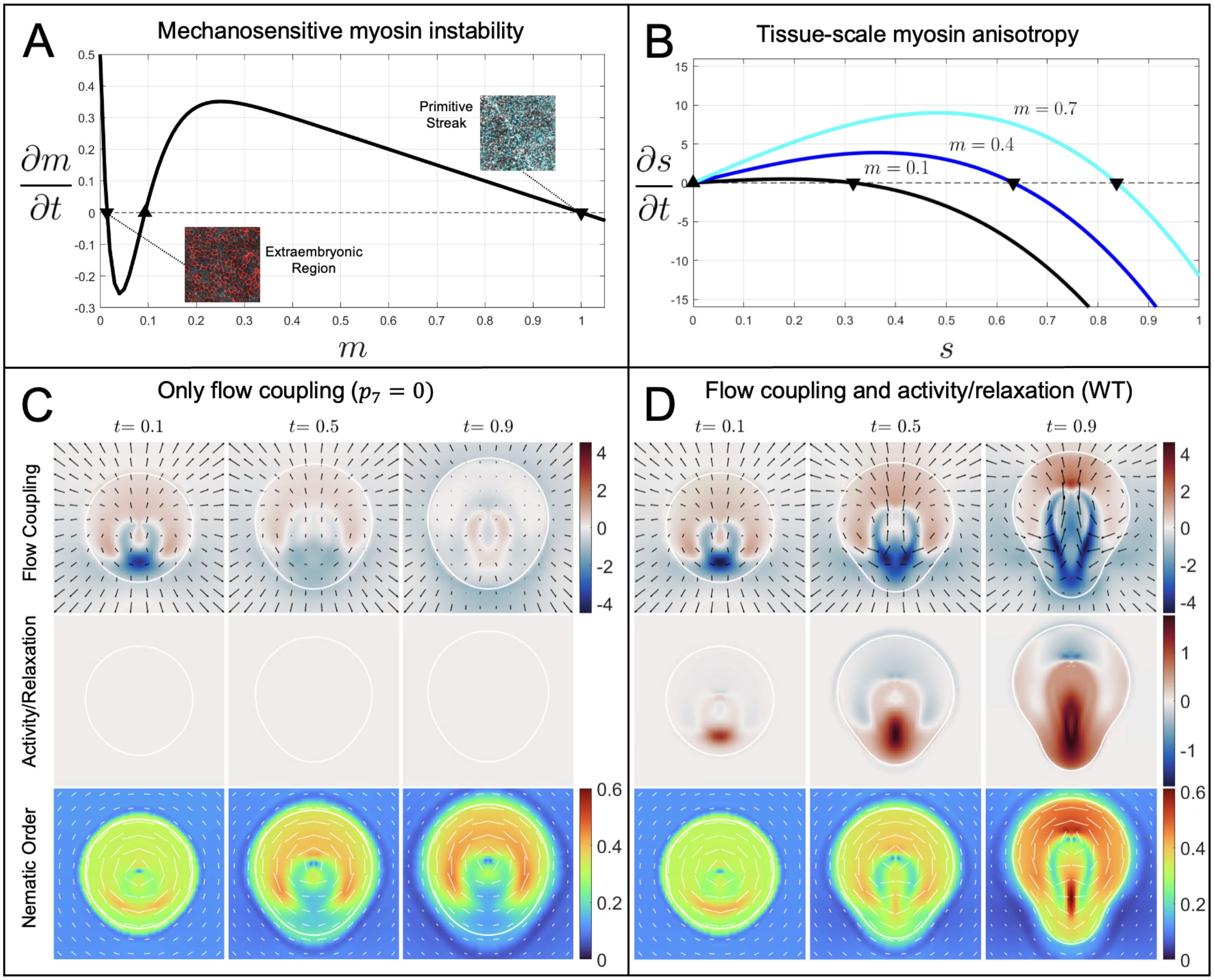

Supplementary Figure S3: **Active Stress Dynamics**: A) Spatially uniform equilibria of active myosin (m) dynamics (Eq. (S10b)) reveal an unstable fixed point (▲) and a low (high) stable fixed points (▼) towards which EE (EP) regions evolve over time [12]. Insets show actin (red) and doubly phosphorylated myosin light chain (cyan) in the EE and primitive streak regions of an embryo after 15 hours (cf. Fig. S9), depicting tissue states closest to the stable equilibria. B) The flow-free ($\mathbf{v} = \mathbf{0}$) equilibria of active myosin nematic order (s) dynamics (Eq. (S5b)) include an instability at $s = 0$ (▲) for $m > 0$ and a higher stable equilibrium (▼) that increases with m. C) Without active alignment and relaxation ($p_7 = 0$), the total derivative of s is governed solely by the flow coupling term (first row). The initially elevated nematic order in the posterior is destroyed by the initial convergent extension (third row), and convergent extension does not continue. D) Inclusion of active alignment and relaxation (middle row, $p_7 = 25$) rescues nematic order from convergent extension, enabling primitive streak formation.

apparent decomposing Eq. (S4) into the separate dynamics of $\mathbf{Q}$'s independent degrees of freedom, ϕ and s [33]:

$$\partial_t \phi = -\overbrace{[\mathbf{v}\cdot\boldsymbol{\nabla}]\phi}^{\text{advection}} + \overbrace{\frac{1}{2}[\cos 2\phi(u_y + v_x) + \sin 2\phi(v_y - u_x)]}^{\text{shear rotation}} + \overbrace{\frac{1}{2}(v_x - u_y)}^{\text{rigid rotation}}, \tag{S5a}$$

$$\partial_t s = -\underbrace{[\mathbf{v}\cdot\boldsymbol{\nabla}]s}_{\text{advection}} + \underbrace{s[\cos 2\phi(u_x - v_y) + \sin 2\phi(u_y + v_x)]}_{\text{flow coupling (shear)}} + s[\underbrace{\gamma m}_{\text{active alignment}} - \underbrace{\delta s^2}_{\text{passive relaxation}}]. \tag{S5b}$$

Here, u_x, u_y, v_x, and v_y denote spatial derivatives of u and v with respect to cartesian coordinates x and y. We use same notation hereafter for all spatial derivatives. Equation (S5a) is the same as in [12], modelling the passive reorientation of cables by shear and vorticity. Instead, Eq. (S5b) is new and accounts for advection, local alignment or destruction of nematic order by shear, active alignment and passive relaxation. Active alignment (γ) in the nematic order dynamics accounts for observations that actomyosin cable formation involves positive feedback, with contracting junctions actively aligning neighboring junctions and anisotropic junctional tension polarizing myosin activity by redistributing myosin to junctions aligned with the direction of highest active stress [14, 18, 21, 24, 34–36]. Relaxation accounts for the combination of non-directed intercalations and resistance to cell shape deformations, which decrease junctional alignment and favor an isotropic tissue state [22, 35, 36]. The quadratic form is typical in nematic models, but a linear form also suffices (Table 3M). Without shearing, active alignment will resist relaxation to achieve a nonzero equilibrium nematic order ($s = \sqrt{\gamma m/\delta}$, Fig. S3B).

S2.3 Force Balance

The total tissue stress σ includes active (σ_A) and viscous (σ_V) components:

$$\sigma = \sigma_A + \sigma_V, \quad \sigma_A = m[\alpha \mathbf{I} + \beta \mathbf{Q}], \quad \sigma_V = 2\mu \mathbf{D}_d + \zeta \boldsymbol{\nabla} \cdot \mathbf{v} \mathbf{I}, \tag{S6}$$

where μ and ζ denote the shear and bulk viscosities, and α and β convert m to isotropic and anisotropic stresses. The anisotropic active stress term arises from the nematic contractile activity of actomyosin cables. The isotropic active stress term instead represents *i)* the apical constriction of cells by contractile activity of myosin in bulk across a cell's apical cortex or in bundles along a cell's perimeter [37] and *ii)* stresses induced by active cell ingressions [12]. Due to the slow flow rates and high viscosity of epithelial dynamics at long times [38], we ignore inertial terms. This leads to an active, compressible Stokes flow ($0 = \boldsymbol{\nabla} \cdot \sigma$):

$$\mathbf{0} = \alpha \boldsymbol{\nabla} m + \beta \boldsymbol{\nabla} \cdot (m\mathbf{Q}) + 2\mu \Delta \mathbf{v} + \zeta \boldsymbol{\nabla}[\boldsymbol{\nabla} \cdot \mathbf{v}], \tag{S7}$$

with x and y components:

$$\begin{bmatrix} 0 \\ 0 \end{bmatrix} = \alpha \begin{bmatrix} m_x \\ m_y \end{bmatrix} + \frac{\beta}{2} \begin{bmatrix} \cos 2\phi \ (ms_x + sm_x + 2ms\phi_y) + \sin 2\phi (ms_y + sm_y - 2ms\phi_x) \\ \cos 2\phi \ (2ms\phi_x - ms_y - sm_y) + \sin 2\phi (2ms\phi_y + ms_x + sm_x) \end{bmatrix} + \mu \begin{bmatrix} u_{yy} + u_{xx} \\ v_{xx} + v_{yy} \end{bmatrix} + \zeta \begin{bmatrix} u_{xx} + v_{yx} \\ u_{xy} + v_{yy} \end{bmatrix}. \tag{S8}$$

S2.4 Analysis in 1D and Biophysical Constraints on α and β

To illustrate the importance of active alignment in Eq. (S5b), we consider a fixed average cable orientation either along ($\phi(x,t) = 0$) or perpendicular to ($\phi(x,t) = \frac{\pi}{2}$) the x-axis and uniform in y. Neglecting the shear force density $2\mu\Delta\mathbf{v}$, the viscous force density simplifies to $\zeta\boldsymbol{\nabla}[\boldsymbol{\nabla} \cdot \mathbf{v}]$ (ζu_{xx} in x), relating to divergence or convergence along x. This reduces the force balance to

$$\phi(x) = 0: \quad 0 = \zeta u_{xx} + \alpha m_x + \frac{\beta}{2}(sm_x + ms_x), \tag{S9a}$$

$$\phi(x) = \frac{\pi}{2}: \quad 0 = \zeta u_{xx} + \alpha m_x - \frac{\beta}{2}(sm_x + ms_x). \tag{S9b}$$

We now ask: given an initial distribution of s or m with maxima centered at $x = 0$, will a negative velocity divergence be induced at $x = 0$ (as in the primitive streak)? If cables contract along x ($\phi = 0$), a peak in m or s will unconditionally induce negative divergence at $x = 0$ (Eq. (S9a)), consistent with the cables' initial tangential orientation resulting in PS formation perpendicular to the cables. However, if cables contract perpendicular to the x-axis ($\phi = \frac{\pi}{2}$), the isotropic (α) and anisotropic (β) effects of myosin activity compete and their contribution to negative velocity divergence becomes conditional. To explore these conditions, we consider two scenarios for which Eq. (S9b) simplifies:

1. Uniform m and locally elevated $s(x)$: $0 = \zeta u_{xx} - \frac{\beta}{2} m s_x$. Here, gradients in s and u_x are always in the same direction, resulting in no PS formation because divergence becomes more positive as s increases.

2. Uniform s and locally elevated $m(x)$: $0 = \zeta u_{xx} + (\alpha - \frac{\beta}{2} s) m_x$. Divergence becomes more negative as m increases only if $s < \frac{2\alpha}{\beta}$ (isotropic active stresses dominate). Since $s \leq 1$, this condition is always met if $\alpha \geq \frac{1}{2}\beta$.

With our analysis, we choose $\beta = \alpha$, satisfying both of the above conditions and reducing parameters.

The 1D model also offers insights into nematic order dynamics in the avian embryo. For cables aligned perpendicular to the primitive streak ($\phi(x) = 0$), the dynamics of s (Eq. (S5b)) becomes $\partial_t s + u s_x = s\big[u_x + \gamma m - \delta s^2\big]$. If $s_x = 0$ (uniform) or $u = 0$ (e.g. in the streak by symmetry), $\partial_t s = 0$ requires $s_{eq} = \sqrt{(\gamma m - u_x)/\delta}$. This highlights two competing effects on nematic order: *i)* active cable alignment and *ii)* nematic order destruction by converging flows along the cable orientation, which can be induced by aligned cables. Active alignment can save nematic order if the active alignment rate exceeds any negative divergence induced. These insights explain why, in the 2-dimension wild-type model, convergent extension cannot be sustained with flow coupling alone (Fig. S3C) but can be saved by active alignment (Fig. S3D). To further simplify the model, we set $\delta = \gamma$, assuming that alignment and relaxation occur on similar timescales.

S2.5 Dimensionless 2D Model

We nondimensionalize Eqs. (S3,S4,S7) using a characteristic lengthscale $x_c = 4\ mm$, representing the radius of the modeling domain that includes the EP and a fraction of the EE region, and a characteristic timescale $t_c = 15\ h$, representing the duration of gastrulation (HH1-HH4). This provides a dimensionless velocity $\mathbf{v}\frac{t_c}{x_c}$ and dimensionless operators $t_c\partial_t$, $x_c\boldsymbol{\nabla}$, and $x_c^2\Delta_{\mathbf{Q}}$. Rearranging terms, we construct a minimal set of dimensionless parameters: $p_1 - p_7$, defined in Table 4. The full 2D model with dimensionless $\mathbf{v}$, m, and $\mathbf{Q}$ becomes

$$\begin{cases} \mathbf{0} = p_1\left[\boldsymbol{\nabla} m + \boldsymbol{\nabla}\cdot(m\mathbf{Q})\right] + p_2\Delta\mathbf{v} + \boldsymbol{\nabla}[\boldsymbol{\nabla}\cdot\mathbf{v}] & \text{(S10a)} \\ \partial_t m = -\mathbf{v}\cdot\boldsymbol{\nabla} m + p_3\left[1 - \left(1 + p_4 e^{-p_5 m}\right) m\right] + p_6\Delta_{\mathbf{Q}} m & \text{(S10b)} \\ \partial_t \mathbf{Q} = -\mathbf{v}\cdot\boldsymbol{\nabla}\mathbf{Q} + s\mathbf{D}_d + \mathbf{W}\mathbf{Q} - \mathbf{Q}\mathbf{W} + p_7\left(m - s^2\right)\mathbf{Q}. & \text{(S10c)} \end{cases}$$

**Dimensionless Model Parameters**	Definition	Value
Active stress to viscous bulk stress	$p_1 = \frac{\alpha t_c}{\zeta}$	4
Shear to bulk viscosities	$p_2 = \frac{\mu}{\zeta}$	0.015
Characteristic timescale to tissue-scale activation timescale	$p_3 = \chi_1 t_c$	0.5
Tissue-scale deactivation to activation rates	$p_4 = \frac{\chi_2}{\chi_1}$	100
Myosin mechanosensitivity	$p_5 = \chi_3$	25
Tension-induced active stress propagation to advective transport	$p_6 = \frac{\epsilon t_c}{x_c^2}$	0.001
Characteristic timescale to active alignment and passive relaxation timescales	$p_7 = \gamma t_c$	15

Table 1: Dimensionless model parameters, their definitions, and default values.

S2.6 Boundary Conditions

Experiments show a nearly linear initial expansion of the outer extraembryonic radius $R_{EE}(t)$ [39,40], consistent with a constant edge cell crawling speed v_e. To model this constant velocity, we use a Dirichlet boundary condition at the outer radius (R) of our circular fixed modeling domain: $\mathbf{v}_R(\theta, t) = [v_R(t)\cos\theta, v_R(t)\sin\theta]$ (Fig. S4A). Without active stress gradients, constant crawling results in a linearly increasing radial velocity profile $v_r(t) = v_e(r/R_{EE}(t))$ so that $v_R(t) = v_e R/(R_{EE}(t_0) + v_e(t - t_0))$ (Fig. S4B). While this velocity boundary condition accurately represents epiboly on our fixed modeling domain boundary, our results are robust to changes in $v_R(t)$ and can also predict observations imposing a constant v_R (Sec. S3). As in [12], the most natural and least intrusive boundary condition for m and $\mathbf{Q}$ is no flux, as experiments suggest no additional sources or sinks of these variables in the EE.

S2.7 Initial Conditions

Consistent with experiments [12,14,21], we model higher initial myosin in the posterior EP with a Gaussian function above a background level $m_> = m^* + 0.001$ (Fig. S3A and Fig. 2A), positioned ($r_c = 0.25$) between the EP center

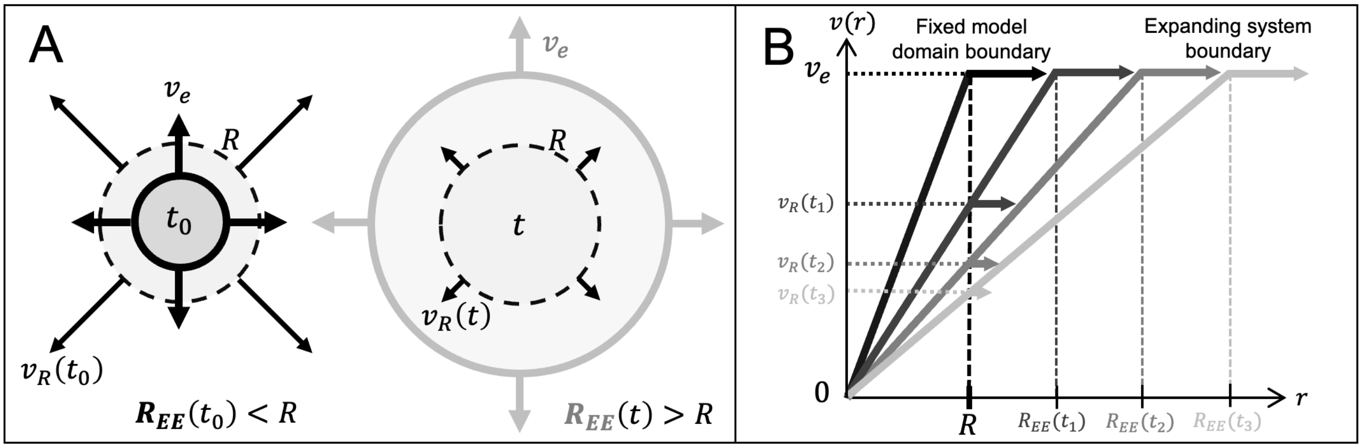

Supplementary Figure S4: **Epiboly-Based Velocity Boundary Condition.** A) Outer extraembryonic radius R_{EE} and model domain radius $R = x_c$ at initial and final times. Initial $R_{EE} < R$ ensures that enough extraembryonic material remains in the domain by final time t to visualize both repellers. B) Constant outward edge-cell velocity magnitude v_e results in a linear velocity profile $v(r)$ and therefore a decreasing domain boundary velocity magnitude $v_R(t)$.

and initial EP boundary ($R_{EP}(t_0) = 0.4$). The Gaussian amplitude $A_m = 0.25$ affects the pace of gastrulation. Its angular and radial extents $\sigma_{r,\theta}$ =0.03, 0.2 reflect the sickle shape of the presumptive mesoderm at HH1. In the EE, where myosin activity starts and remains low, we initialize myosin $m_<$ below the instability. We set the initial orientation of actomyosin cables $\phi(\mathbf{x}, t_0)$ along the tangential direction, matching experimental observations [12]. We set the initial nematic order $s(\mathbf{x}, t_0)$ to its equilibrium value in the absence of flow, determined by active alignment, passive relaxation, and the initial distribution of myosin activity (Fig. S3B). This choice is justified by the observed initial anisotropy in cell shapes in the posterior EP, even before the onset of motion [14]. Equation (S10a) determines the velocity at each instant, given m and $\mathbf{Q}$ with boundary velocity $\mathbf{v}_R$. We nondimensionalize r_c, σ_r, $R_{EP}(t_0)$, and v_e using x_c and t_c. The dimensionless effective edge cell velocity $v_e/R_{EE}(t_0) = 0.5$ is consistent with the typical epiboly velocity observed in our experiments ($50 - 150\ \mu m/h$). We use these initial conditions, summarized in Table 2, together with Eq. (S10), to simulate gastrulation over 15 hours (stages HH1-HH4).

**Model Variables**	Initial Condition	
Myosin Intensity	$m_0 = \begin{cases} m_> + A_m e^{-\frac{(r-r_c)^2}{2\sigma_r^2} - \frac{(\theta)^2}{2\sigma_\theta^2}} \\ m_< \end{cases}$	for $\mathbf{x}_0 \in \mathbf{x}_{EP}$, i.e. $0 < r \leq R_{EP}(t_0)$<br>for $\mathbf{x}_0 \in \mathbf{x}_{EE}$, i.e. $R_{EP}(t_0) < r \leq R$
Myosin Orientation	$\phi_0 = \theta + \frac{\pi}{2}$	
Myosin Anisotropy	$s_0 = \sqrt{m_0}$	

Table 2: **Model initial conditions**. Variables are defined in polar coordinates $r = \sqrt{x^2 + y^2}$ and $\theta = \arctan y/x$ computed from the Cartesian coordinates $\mathbf{x} = [x, y]$ on a circular modeling domain centered at $\mathbf{x} = \mathbf{0}$.

S2.8 Numerical Scheme

We solve Eq. (S10) in MATLAB using a finite-difference numerical scheme on a circular domain (Algorithm 1). To compute advection terms, we use first-order upwinding to ensure stability and avoid introducing spurious oscillations. Near the EP-EE boundary, where there are steep gradients in m and $\mathbf{Q}$, we instead use second-order upwinding to minimize numerical diffusion. We discretize all other spatial differential operators using second-order centered finite differences, except at the boundaries, where we use first-order forward or backward finite differences [41].

Algorithm 1 Numerical Solver of Eq. (S10)

Inputs:
Numerical Parameters: Spatial discretization $\Delta x = 0.0125$ and temporal discretization $\Delta t = 0.001$.
Grid: $x_i = i\Delta x,\ y_j = j\Delta x,\ \forall\ i, j$ such that $\sqrt{i^2 + j^2} \leq N,\ N\Delta x = 1$.
Initial Conditions: $m_0,\ \phi_0,\ s_0,\ t = t_0 = 0$.
Boundary Conditions: $\mathbf{v}_R$.
Model Parameters: $p_1, .., p_7$.

1. Solve for $u(x, y, t_k)$, $v(x, y, t_k)$ using Eq. (S10a).
2. Compute $m(x, y, t_{k+1})$, $\mathbf{Q}(x, y, t_{k+1})$ using Eqs. (S10b-S10c) with $v(x, y, t_k)$, $u(x, y, t_k)$, $m(x, y, t_k)$, and $\mathbf{Q}(x, y, t)$ and Euler's method.
3. Update boundary condition $v_R(t)$ to reflect continued edge cell migration (cf. Sec. S2.6).
4. Repeat steps 1-3 for all $t_k = k\Delta t,\ k = 0, .., N_t,\ N_t\Delta t = 1$.

Outputs: u, v, m, and $\mathbf{Q}$ at all grid points $\{x_i, y_j, t_k\}$.

S3 Model Perturbations

In the main text, we present only model perturbations that are experimentally realizable. To confine the simulated embryo, we blocked epiboly ($v_e = 0$), resulting in a fixed embryonic radius ($v_R(t) = 0$). This change dramatically weakens R1 compared to the wild type. Complete elimination requires eliminating isotropic myosin activity in the EP (Table 3A). Solely eliminating isotropic myosin activity also eliminates R1 (Table 3B), but this is not experimentally realizable without also affecting anisotropic myosin activity [14] (eliminating both repellers). Likewise, the distinct myosin dynamics of the EP and EE are necessary for Repeller 1 (Table 3C). R2 bisects the presumptive mesendoderm region, which, in the model, is characterized by higher initial myosin activity. Accordingly, in the main-text model perturbation, we eliminated the extra posterior myosin ($A_m = 0$), making the model radially symmetric. While this alone is sufficient to eliminate R2, it can also be achieved by blocking active alignment ($p_7 = 0$, Table 3D) or anisotropic myosin activity (enforcing $s = 0$, Table 3E). However, these are not feasible in experiments.

These model perturbations, whose experimental analogs are impractical, help clarify the necessary and sufficient mechanisms for each repeller and their contributions to the embryo's flows and dynamic geometry. Table 3 includes a series of additional perturbations, varying model parameters, boundary conditions, and initial conditions to elucidate the importance of certain model components and the flexibility of their parameters. Increasing (decreasing) p_1 raises (lowers) active stress magnitudes and strengthens (weakens) the repellers (Table 3O-P). p_2, relating shear and bulk viscosities, encodes tissue fluidity. A more fluidized tissue predicts larger vortices and stronger repellers (Table 3F). Conversely, a less fluid embryo exhibits no large, vortical flows, as previously noted [12], and, crucially, no shape change from circular to pear-shaped (Table 3G). Parameter p_3 scales myosin activity's overall rate of change while p_4 and p_5 shape the curve in Fig. S3A. p_3 must be large enough to produce the observed increase in myosin activity and rapid streak generation, but not too large such that myosin activity saturates in the whole EP within the gastrulation timescale. A five-fold reduction (Table 3Q) or increase (Table 3R) falls between these extremes. The unstable fixed point of m is key to diverging EP/EE dynamics (Table 3L). Large simultaneous decreases (Table 3S) or increases (Table 3T) in p_4 and p_5 both preserve the instability and yield similar results. Finally, a 50% decrease or increase in p_6 (Table 3U-V) or p_7 (Table 3W-X) yields similar results. Faster edge-cell crawling strengthens R1 and enlarges the EP (Table 3H). Using a constant v_R instead of our dynamic $v_R(t)$ simplifies the model without substantially affecting our results (Table 3I), but is less realistic.

Finally, we explore the effects of delaying either the onset of epiboly or posterior myosin elevation (Fig. S13). We find that time delays in edge cell crawling weaken R1 while preserving R2 (Table 3J), and time delays in convergent extension weaken R2 while preserving R1 (Table 3K), consistent with their mechanistic modularity. Notably, substantial EP shape change in the model occurs only when convergent extension starts before epiboly, as typically occurs in experiments.

	**Perturbation**	Effect	Movie
A	No isotropic myosin activity and $v_e = 0$	Eliminates R1	Supplementary Movie 5
B	No isotropic myosin activity	Eliminates R1	Supplementary Movie 6
C	No EE-EP distinction ($m_0 = m_>$ for $r > R_{EP}(t_0)$)	Eliminates R1	Supplementary Movie 7
D	No active alignment ($p_7 = 0$)	Loses convergent extension	Supplementary Movie 8
E	No anisotropic myosin activity ($s = 0$)	Eliminates R2	Supplementary Movie 9
F	Higher fluidity ($p_2 = 0.01$)	Third repeller (see Fig. S8)	Supplementary Movie 10
G	Lower fluidity ($p_2 = 0.5$)	No vortices or shape change	Supplementary Movie 11
H	Stronger epiboly ($v_e = 2$)	EP area enlarged	Supplementary Movie 12
I	Constant boundary velocity ($v_R = 0.4$)	Minimal effect	Supplementary Movie 13
J	Delayed edge cell crawling ($t_{\mathrm{CE}} - t_{\mathrm{EC}} = -0.5$)	Weak R1	Supplementary Movie 14
K	Delayed convergent extension ($t_{\mathrm{CE}} - t_{\mathrm{EC}} = 0.5$)	Weak R2, no shape change	Supplementary Movie 15
L	No Mechanosensitivity ($p_5 = 0$)	Eliminates R1, R2, Attractor	Supplementary Movie 16
M	Linear passive relaxation	Minimal effect	Supplementary Movie 17
N	Line initial condition, no anisotropic myosin activity	Attractor without R2	Supplementary Movie 18
O	Decreasing p_1 50%	Minimal effect	Supplementary Movie 19
P	Increasing p_1 50%	Minimal effect	Supplementary Movie 20
Q	Decreasing p_3 50%	Minimal effect	Supplementary Movie 21
R	Increasing p_3 50%	Minimal effect	Supplementary Movie 22
S	Decreasing p_4 80%, p_5 68%	Minimal effect	Supplementary Movie 23
T	Increasing p_4 900%, p_5 900%	Minimal effect	Supplementary Movie 24
U	Decreasing p_6 50%	Minimal effect	Supplementary Movie 25
V	Increasing p_6 50%	Minimal effect	Supplementary Movie 26
W	Decreasing p_7 50%	Minimal effect	Supplementary Movie 27
X	Increasing p_7 50%	Minimal effect	Supplementary Movie 28

Table 3: **Model perturbations**. Associated movies depict the model velocities, velocity divergence, isotropic and anisotropic stresses, active forces, repellers, attractors, and deformed Lagrangian grids over time.

S3.1 Active Forces

Supplementary Movies 1-28 include the magnitude and direction of active forces $p_1[\boldsymbol{\nabla} m + \boldsymbol{\nabla} \cdot (m\mathbf{Q})]$ (cf. Eq. (1a)), indicating, in addition to the boundary velocity, the active drivers of the gastrulation flows. Active forces are strong around the presumptive mesendoderm and later in the vicinity of the developing streak consistent with our earlier work [12]. These active forces, arising from the active intercalation and ingression of mesendoderm cells, generate EP vertical flows, convergent extension and shape change. In addition, the gradient in myosin activity between the EE and EP results in inward active forces constricting the EP at its boundary. These active forces largely depend on isotropic myosin activity and generate Repeller 1 (Table 3B) but are insufficient to drive gastrulation flows and embryo shape change alone (Table 3E). In fact, removing the differential EE-EP myosin dynamics eliminates this second pattern of active forces (Table 3C).

S3.2 Connection Between R2 and the Attractor

R1 and R2 arise from distinct mechanisms and can be experimentally separated. In contrast, R2 and the Attractor are inseparable in chick, as they are generated by convergent extension and ingression of the same mesendoderm tissue region. From purely kinematic considerations, convergent-extension flows necessarily create an attractor (from convergence) and a repeller (from extension). For example, the simplest convergent-extension flow velocity $\mathbf{v} = [-x, y]$ generates an attractor (y-axis) and repeller (x-axis). In the mesendoderm, additional isotropic convergence associated with cell ingression sharpens the Attractor. Consistent with this argument, inhibiting ingression (Fig. 2J in [42]) using the vascular endothelial growth factor (VEGF) receptor inhibitor axitinib preserves convergent extension motion, but reduces isotropic convergence (Fig. 2K-L in [42]), generating a thicker (i.e., less sharp) attractor and a smaller domain of attraction (Fig. 4C-D in [12]).

Our model predicts that convergent extension arises from anisotropic active forces (generating an attractor, repeller pair). Simultaneously, isotropic active forces associated with ingressions sharpen the Attractor. For these reasons, initializing myosin directly along the midline instead of as a crescent converging to the midline, the model—without anisotropic active forces—generates a line attractor without R2 (Table 3N). This scenario resembles the early mouse embryo, where mesendoderm is pre-patterned along the midline [43], and cells undergo apical contraction and ingression without oriented intercalation. This theoretical demonstration of attractor-repeller separability suggests additional modular potential and offers insights into how different vertebrate species may have evolved distinct gastrulation modes (with or without a repeller) by modifying the relative contributions of isotropic and anisotropic active forces. The presence of R2 in chick may help support compartmentalization [44] by reducing communication between nearby cells separated into the anterior and posterior primitive streak

S4 Note on Epiboly and Evolution

Changes in the amount of yolk in the egg have been recognized as one of the main drivers of evolutionary change during gastrulation [45–47]. Amniotes (reptiles, birds, and mammals) evolved from ancestors that could lay eggs on land, achieved through the acquisition of a mineralized shell that provided mechanical protection and three extraembryonic membranes [48]. Most importantly, the yolk experienced a significant increase in size, probably to increase the egg's energy depot, allowing the embryo to hatch as a miniature version of the adult instead of as a larva [49]. This increase in yolk size generated mechanical and topological constraints that required adaptations in the gastrulation mode.

The early cleavages became exclusively meroblastic due to the impossibility of splitting the enormous yolk cell [48,50], leading to the formation of discoidal embryos that sit on top of the yolk. This topological configuration imposes a problem on the developing embryo, as it now has to engulf the yolk underneath to effectively access its nutrients. Consequently, large yolks are associated with the evolution of extraembryonic tissues that engulf the yolk and later help to digest it, as seen in teleost fish and amniotes. The main role of epiboly, the process by which extraembryonic tissues expand and thin to enclose the yolk, is to form the yolk sac and provide nutrients to the embryo later in development [51].

In amniotes, including chicken, the extraembryonic tissues have acquired an additional role in patterning the embryo during gastrulation. For example, in chick, FGF8 and WNT9C secreted by the the hypoblast and the EE help to position the mesoderm [52]. However, the importance of the mechanical inputs imposed by the EE on the embryo proper (EP) and their significance for development remains an open question. Epiboly induces global tension [39, 40, 51, 53] that propagates to the EP, as severing the EP-EE boundary causes both regions to contract [51]. Previous studies have suggested that epiboly is necessary for correct early development in avian embryos, as ablating large EE areas at primitive streak stages leads to poor embryo development [51]. However, it is unclear if this is due to the removal of mechanical inputs or the disruption of signaling roles played by the EE.

Our findings reveal that epiboly is not required for the early stages of avian embryogenesis. When we confined the embryo to eliminate epiboly while maintaining an intact EE, embryos still gastrulated and developed complete axial structures (brain, somites, neural tube, tailbud), despite having shorter, but proportioned, body axes (Fig. S10). Recent reports have shown that increasing tension in the chick embryo results in a shorter body axis [54]. Similarly, disrupting epiboly progression in zebrafish does not prevent gastrulation but produces a shorter body axis [55, 56]. These findings indicate that while epiboly movements are not required for gastrulation to occur, they can impact embryo body length at later stages.

This suggests that the evolution of the amniote egg likely necessitated the development of mechanisms to maintain the shape and integrity of the EP during epiboly. Embryo contractility in amniotes may have evolved as a mechanism to resist the influence of epiboly, allowing the embryo to maintain its intrinsic patterning mechanisms and developmental timeline, ensuring proper body plan formation. This idea is supported by experiments showing that partial EP ablations normally require detachment of the EE from the vitelline membrane to avoid the embryo ripping apart [57], suggesting that epiboly forces can pull apart a mechanically compromised EP.

The evolution of the amniote egg, with its increased yolk size and extraembryonic membranes, has led to significant adaptations in the gastrulation process. While epiboly is essential to construct the structure that will provide

nutrients to the developing embryo, our findings suggest that it may not be strictly required for the early stages of avian embryogenesis. In fact, epiboly might even be a developmental burden that the early embryo must overcome. The contractility of the amniote embryo may have evolved as a mechanism to maintain its shape and integrity during epiboly, allowing it to follow its intrinsic developmental program without being excessively influenced by the mechanical forces imposed by the extraembryonic tissues. These insights underscore the complex interplay between mechanical forces, signaling pathways, and evolutionary adaptations in shaping the gastrulation process and embryonic development.

S5 Experimental Methods

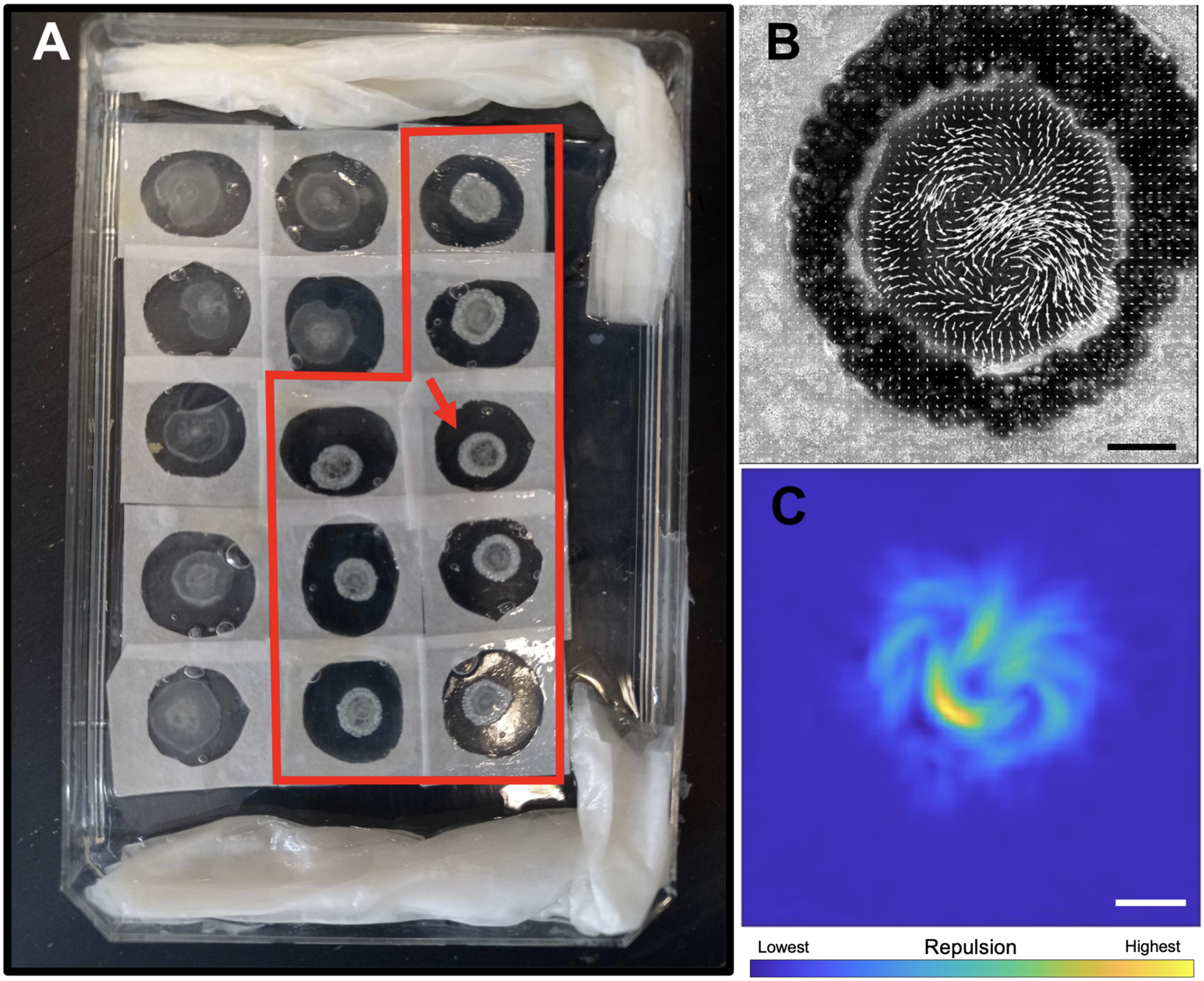

Supplementary Figure S5: **High Throughput Quantification of Chick Gastrulation Flows.** A) Plate of 15 chick embryos culture *ex-ovo* for automatic wide-field time-lapse imaging using a motorized stage. The red polygon delineates confined chick embryos. The arrow indicates the embryo shown in B-E. B) Quantification of tissue flows using particle image velocimetry. Arrows are enlarged for visualization. C) Large values of ${}_2\lambda_{t_0}^{t}(\mathbf{x}_0)$ computed from particle image velocimetry velocities mark repellers (cf. Sec. S1). Scale bars are 1 mm. Supplementary Movie 29 shows the simultaneous development of 8 wild type (top) and 8 confined (bottom) embryos. Each box containing an embryo is 8.3 mm wide.

All the following procedures comply with all relevant ethical regulations.

S5.1 *Ex Ovo* Culture

Fertilised white Leghorn chicken eggs (Medeggs Ltd, Norfolk, UK) were incubated at 37°C for 1-6 hours to around Hamburger Hamilton stage 1 (HH1). Embryos were isolated on a windowed piece of filter paper and cultured *ex ovo* on a semisolid agar-albumen medium, ventral side up [58]. To acquire high-quality velocity fields capturing cell movements on the embryo surface, it is essential to remove as much yolk as possible from the embryos. Embryos were cleaned with a mini Pasteur pipette using a warm 0.1% Tween in physiological saline solution and rinsed with physiological saline solution. For high-throughput live imaging, a large transparent plate was covered with semisolid agar-albumen medium surrounded by wet paper and in the presence of antibiotics, where the embryos were arranged in a grid (Fig. S5A) with the ventral side up.

S5.2 Mechanical Confinement and Chemical Perturbations

To mechanically confine embryos we cultured chick embryos on a piece of filter paper with a wide opening (∼15 mm). Later, we cauterized the embryonic side of the vitelline membrane surrounding the embryo using a soldering iron at 250°C, creating a fixed boundary of denatured material that prevents epibolic expansion. For chemical perturbations, a pan-FGF receptor inhibitor (LY2874455, $1\mu M$, SelleckChem) was added to the culture medium to block mesoderm differentiation as in [42]. To generate a circular PS we carefully deposited a $1\mu L$ drop of $50\mu g/mL$ human FGF2 (provided by the Cambridge Stem Cell Institute, University of Cambridge) on the hypoblast side of the embryo as in [42]. Control embryos were cultured under identical conditions without confinement or in the presence of 0.1% DMSO.

S5.3 Velocimetry

Embryos were imaged with a Nikon Eclipse Ti inverted bright-field microscope equipped with a motorized stage, a 20X objective, and an Orca Flash 4.0 camera. Tile scan images (6x6 or 7x7) were acquired every 30 minutes for up to 24 hours. When using chemical inhibitors, the embryos were imaged in a 6-well plate with individual agar-albumen substrates for each embryo to avoid chemical diffusion to control samples. Velocity fields were computed from time-lapse movies of developing embryos using PIVLab v2.56 for MATLAB with three passes of 576×576, 144x144 and 72x72 pixel interrogation windows with 50% overlap with default pre- and post-processing parameters. Deformation grids, decomposed strain rates, and the DM were then computed from the velocity field as in [1, 14, 42, 59].

S5.4 Determination of Embryo Proper, and Extraembryonic Areas Over Time

To determine the embryo size over time, a polygon is manually drawn around the embryo's edge and tracked using the velocity fields. To obtain the the area of the EP over time the repeller field of the DM (${}_2\lambda_{t_0}^t$) is overlapped on the embryo as a guide to draw a polygon at the EP boundary.

S5.5 Immunohistochemistry

Embryos were fixed in 4% paraformaldehyde overnight at 4°C, permeabilized with PBS - 0.1% Tween (PBT), and blocked with 10% goat serum and 2% bovine serum albumin in PBT. A primary rabbit antibody against double phosphorylated Thr-18 / Ser-19 and myosin light chain 2 (3674, Cell Signaling Technology) was applied overnight at 4°C with 1:50 dilution. After washing, embryos were incubated with an Alexa Fluor™ 555 goat anti-rabbit secondary antibody (1:500 dilution, Invitrogen) in the presence of Alexa Fluor™ Plus 405 Phalloidin (1:1000 dilution Invitrogen) and SYTOX™ Deep Red Nucleic Acid Stain (1:1000 dilution Invitrogen) overnight at 4°C. Finally, embryos were washed, mounted between two thickness 1 cover slides with VECTASHIELD® Antifade Mounting Medium (Vector Laboratories), and imaged using a Zeiss LSM700 confocal microscope with a 20X or a 40X objective and 0.5 zoom.

S5.6 Computational Surface Extraction

Active myosin resides in the most apical compartment of the cells. For this reason, maximum projections may not accurately represent the apical myosin, including the myosin cables, across the embryo. To tackle this problem we computationally acquire a 2D surface of the most apical side of the embryo. To extract the apical surface of the

embryo from the confocal image volumes, we used a custom MATLAB script based on the square gradient focusing algorithm [59, 60]. The steps are as follows:

1. Tile the confocal volume into columns.
2. Apply the square gradient focusing algorithm to each column to find the surface position.
3. Generate a height map by finding the depth of the fastest change in the image sharpness (peak of the second derivative) for each column.
4. Apply a smoothing filter to the height map.
5. Use the smoothed height map to section the image volume and produce a 2D image of the embryo surface.

S5.7 Statistics and Reproducibility

For the analysis of tissue flows we used 8 embryos per group for control and confined conditions, and 4 embryos per group for treatments with LY2874455/FGF2 and confined with LY2874455/FGF2. The immunostained images shown in Figs. 2E, 3D, 4D, and S9 are representative of 4, 3, 4 and 3 embryos, respectively. For the analysis of the confinement treatment (Fig. S10) we used 8 embryos per condition. We compared the results using MATLAB's implementation of the Two-sample t-test (ttest2).

S6 Additional Supplementary Figures & Tables

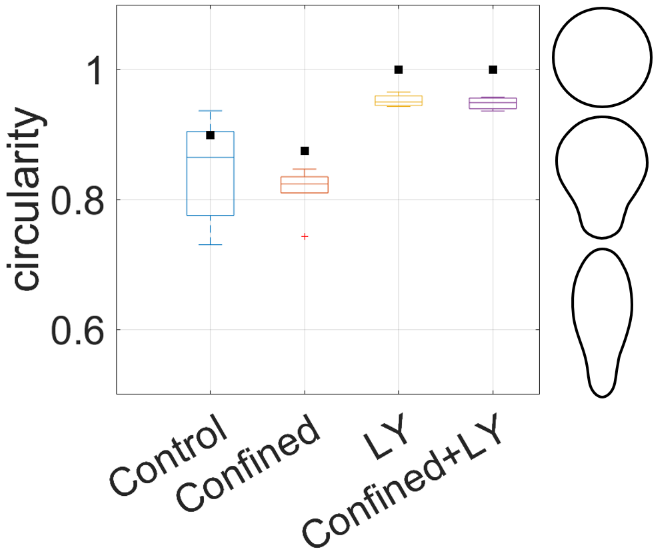

Supplementary Figure S6: **Shape quantification.** EP's circularity ($4\pi(\text{area})/(\text{perimeter})^2$) in different treatments. Black squares mark EP circularity in model simulations. Control (Fig. 2), Confined (Fig. 3), LY (Fig. 4), LY + confined (Fig. S11D). LY2874455 $1\mu M$, $N = 8, 8, 4, 4$, respectively.

**Parameter**	In [12]	Connection
—	p_0	In this paper, we do not separately model ingression.
p_1	—	Converts active myosin fraction m to dimensionless active stress (Sec. S2).
p_2	p_1	Lower effective shear viscosity needed for dynamic EP geometry.
—	p_2	Rod-like nematic elements ($= 1$) assumed.
$p_3 - p_5$	$p_3 - p_5$	To match shape of Fig. S3A with shape of Fig. S3A in [12].
—	p_6	m saturation now imposed by the form of Eq. S10b.
p_6	p_7	Larger domain size (x_c).
p_7	—	New parameter for s dynamics (Sec. S2.2).

Table 4: **Parameter comparison with [12].** Some corresponding parameter values have changed i) due to the expanded EP/EE domain, ii) to allow EP shape change and iii) due to the redefinition of m as a dimensionless fraction instead of a dimensionless stress.

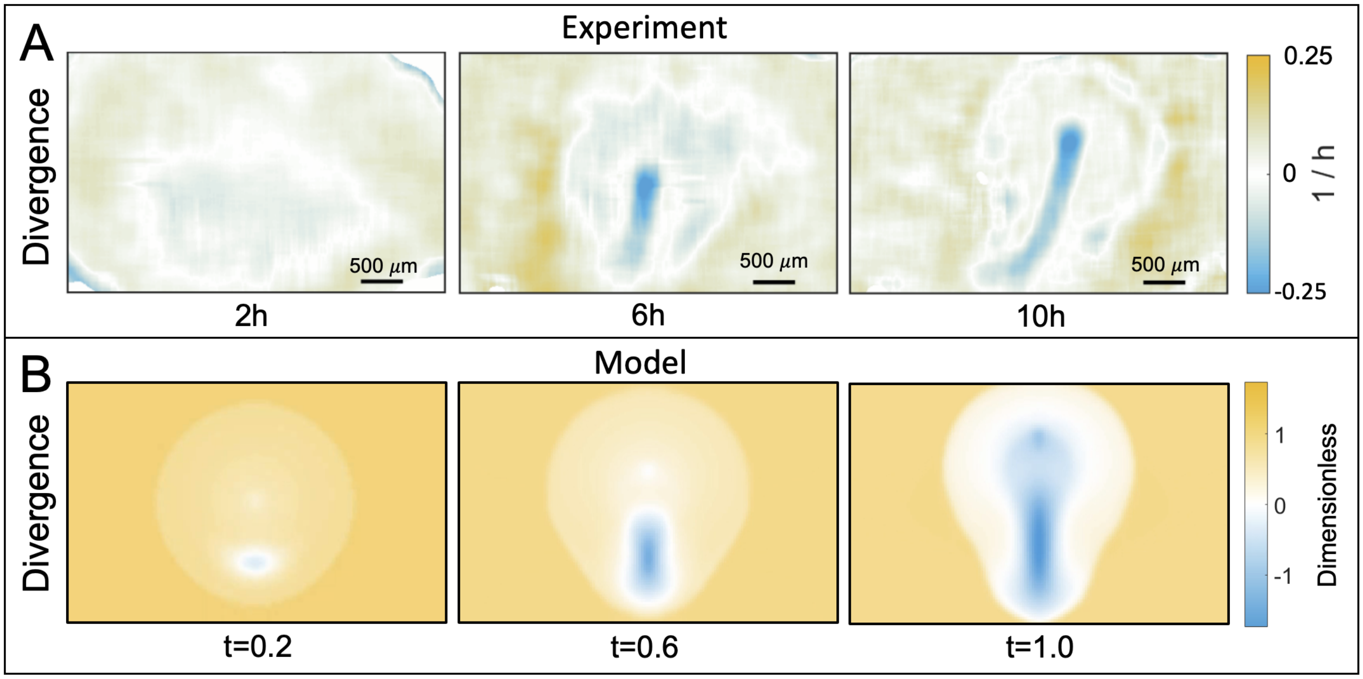

Supplementary Figure S7: **Velocity Divergence.** A) Velocity divergence $\nabla \cdot \mathbf{v}$ in wild-type experiments (A) and model (B) at three times. In A, 0 h corresponds to HH1.

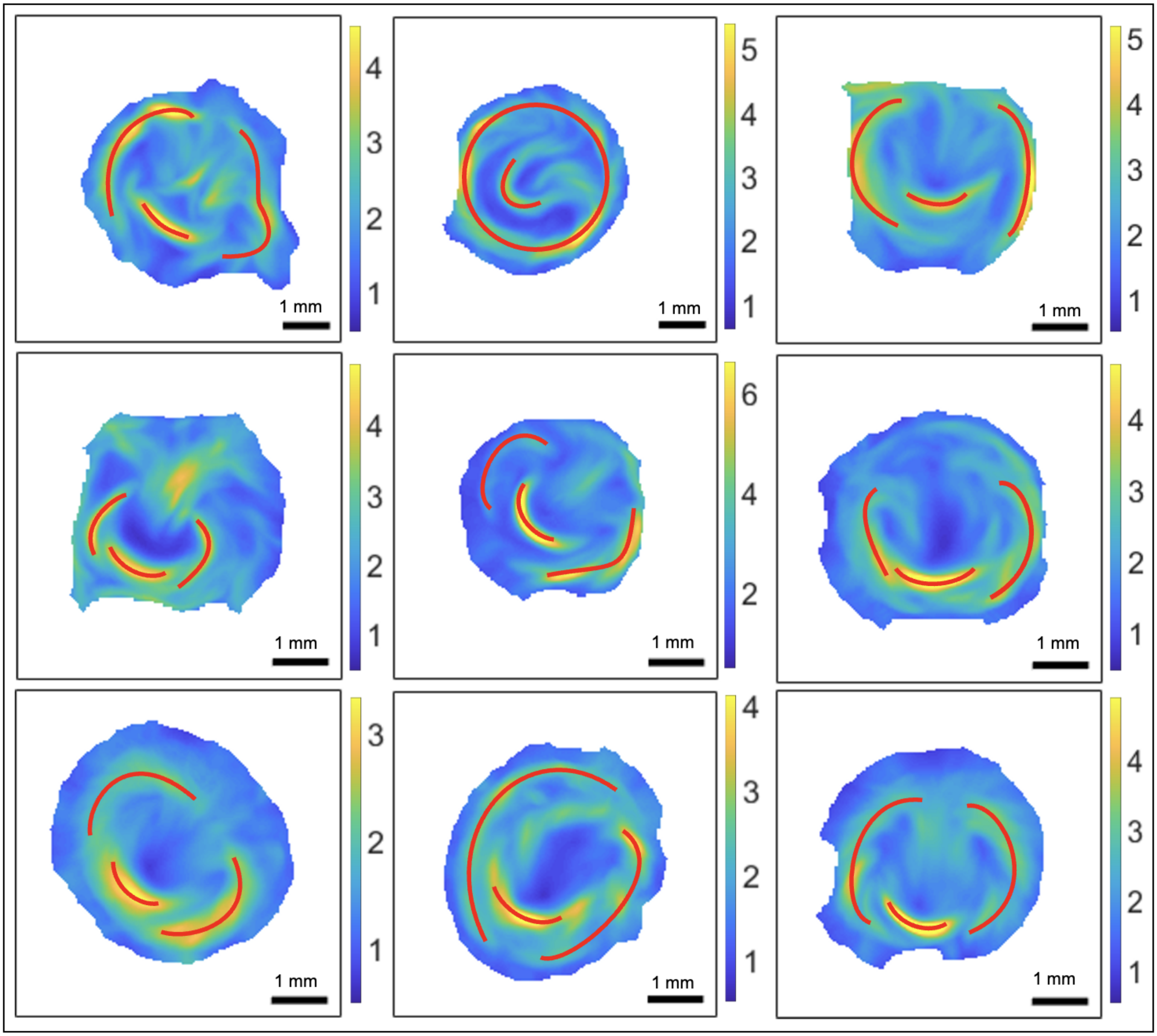

Supplementary Figure S8: **Robustness and Intrinsic Variability of Repellers.** Experimental unnormalized ${}_2\lambda_{t_0}^{t}(\mathbf{x}_0)$ for 9 wild-type embryos recorded for $\approx 15\ h$. Red curves mark the repellers (R1 and R2). Each repeller's precise shape and position vary, but R1 consistently wraps around the initial EP boundary while R2 consistently arcs across the posterior. We note the variability in the intensities of R1 and R2 as well as the occasional appearance of a third repelling structure in the anterior, associated with lateral separation (perpendicular to the AP axis) of cells at opposite sides of the PS when it approaches the anterior EP boundary. This feature is also present in the model repeller field (Fig. 2H) and visualizable from the co-located tangential deformation in the Lagrangian grid (Fig. 2F). Note the robustness of repellers and the DM despite our high throughput experimental approach using a bright-field microscope (Sec. S5) generating less resolved tissue flows compared to single-embryo experiments from a light-sheet microscope (cf. Fig. 3A in [1]). Supplementary Movie 29, top shows the simultaneous development of 8 wild-type embryos. Each box containing an embryo is 8.3 mm wide.

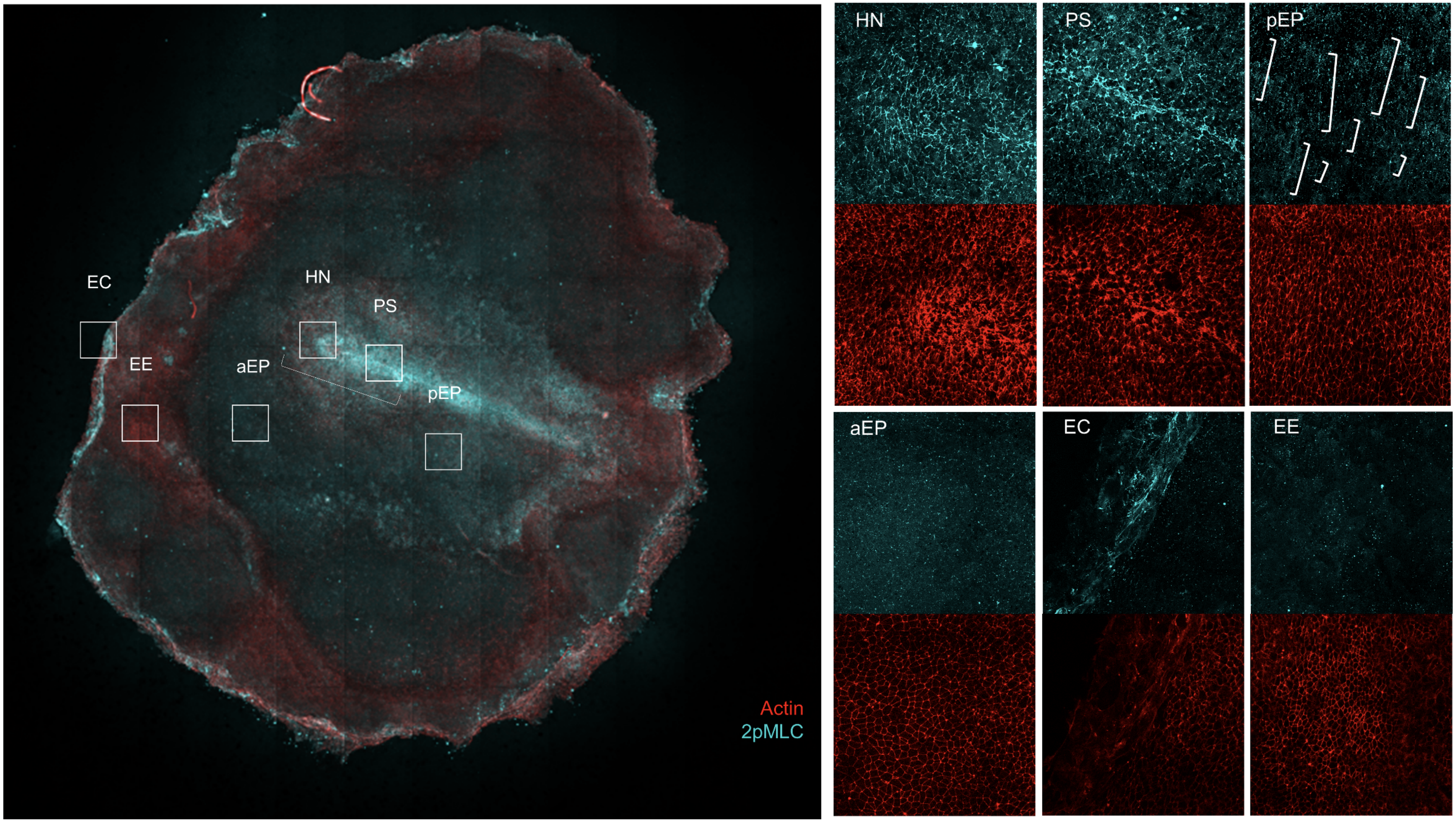

Supplementary Figure S9: **Characterisation of Myosin Patterns in a Confined Embryo.** Overview) Full image of a confined embryo at stage HH3+. Each box is 400 μm in side. HN) Hensen's node, PS) Primitive streak, pEP) Posterior embryo proper, note long myosin cables perpendicular to the PS aEP) Anterior Embryo proper, EC) Edge cells, EE) Extraembryonic territory.

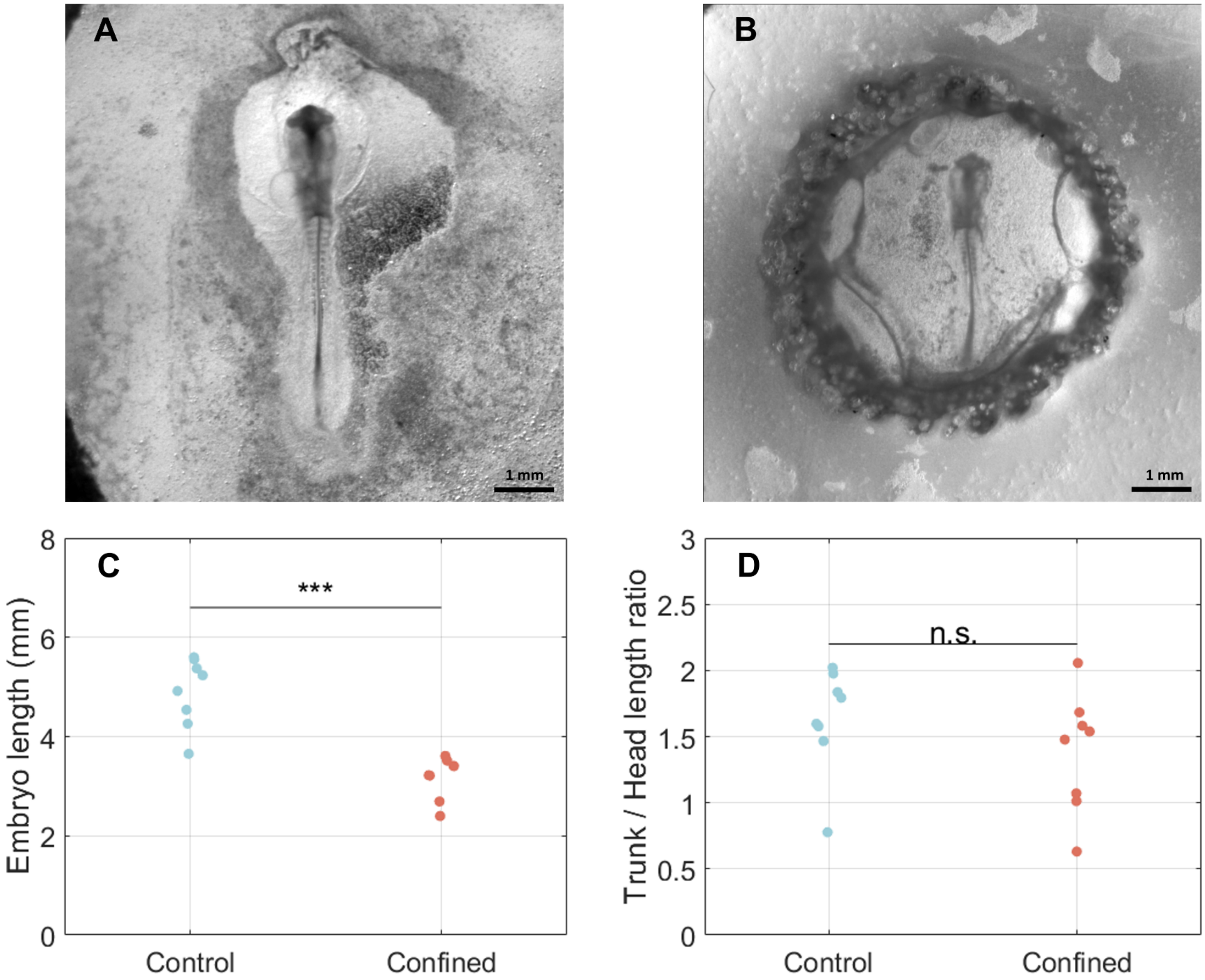

Supplementary Figure S10: **Embryos Are Shorter but Proportional After 48 Hours of Confined Development.** A) Embryo after 48 hours of *ex-ovo* development. B) Confined embryo after 48 hours of *ex-ovo* development. C) Embryo length for control and confined embryos after 48 hours of development (p-value=$7.67 \cdot 10^{-5}$). D) Trunk/head length ratio for control and confined embryos after 48 hours of development (p-value=0.26). N=8, Statistical analysis: Two-sample t-test, p-value<0.001 (***), p-value>0.05 (not-significant, n.s.).

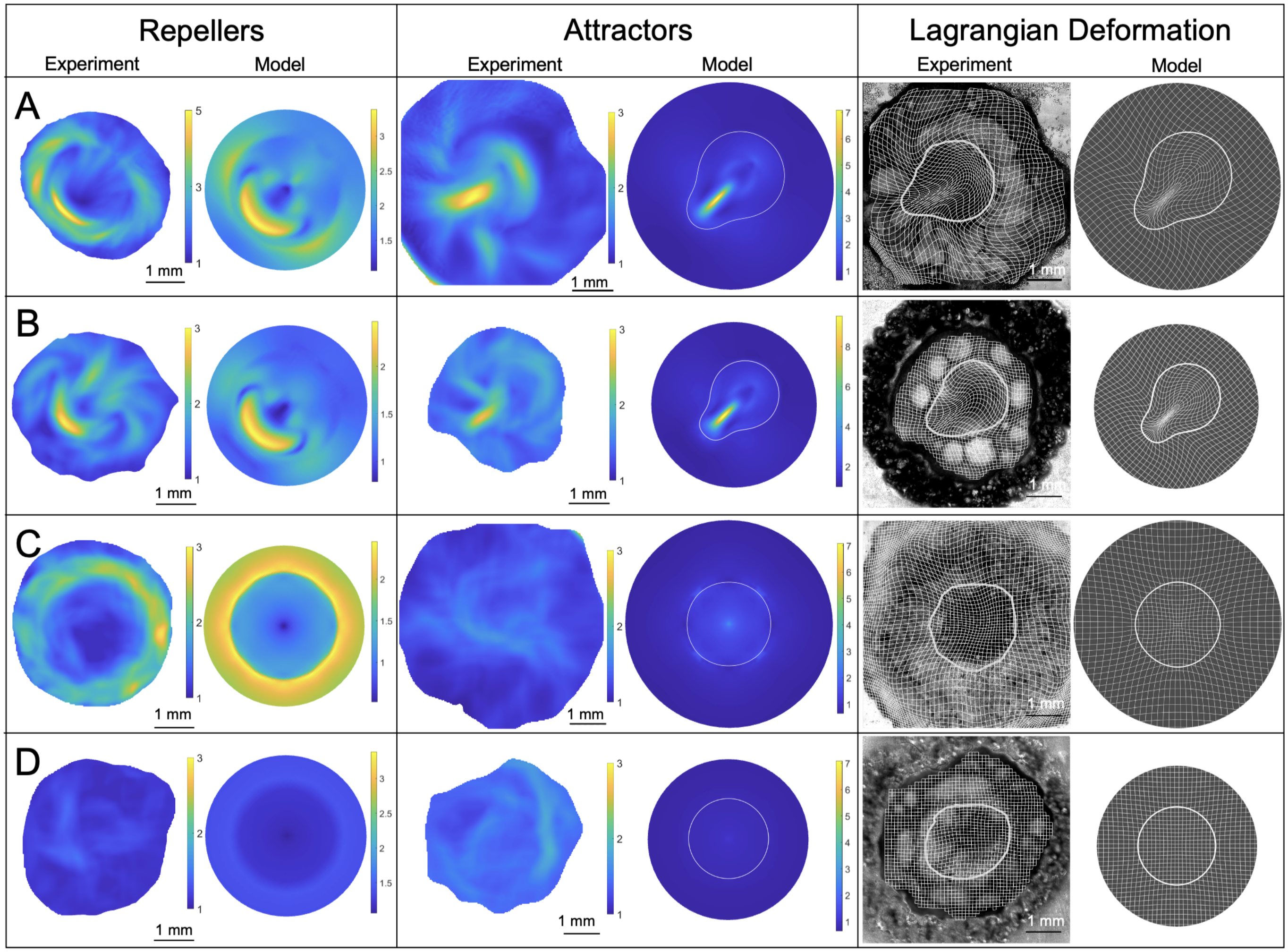

Supplementary Figure S11: **Dynamic Morphoskeleton in Main-Text Perturbations.** Repellers, attractors and Lagrangian deformation for combinatorial elimination of R1 and R2. The Attractor marks the PS (cf. Fig. 1). White curves mark the EP boundary at final times t_f. A) Wild type, both repellers present. B) Confined, R1 eliminated. C) No mesoderm, R2 eliminated. D) Combined, both repellers eliminated. Eliminating R2 eliminates the Attractor. ${}_2\lambda_{t_0}^{t_f}(\mathbf{x}_0)$ (${}_2\lambda_{t_f}^{t_0}(\mathbf{x}_f)$). The repeller field in C and repeller and attractor fields in D use the WT colorbar (A) to emphasize the relative lack of deformation.

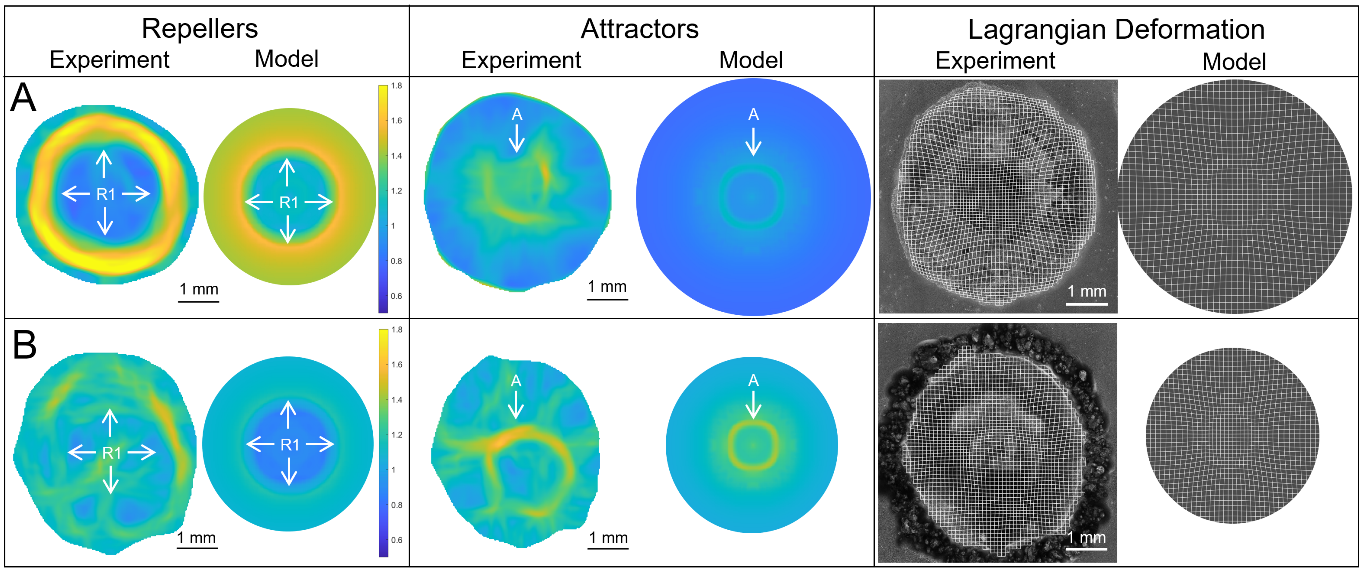

Supplementary Figure S12: **Circular Mesoderm.** Repellers, attractors and Lagrangian deformation with circular mesoderm (FGF treatment) in unconfined (A) and confined (B) conditions. Colorbars for repellers (attractors) mark ${}_2\lambda_{t_0}^{t_f}(\mathbf{x}_0)$ (${}_2\lambda_{t_f}^{t_0}(\mathbf{x}_f)$). Circular mesoderm is modeled by replacing the crescent-shaped myosin initial condition with a radially symmetric Gaussian function with radius 0.25, standard deviation 0.03, and amplitude $A_m = 0.15$.

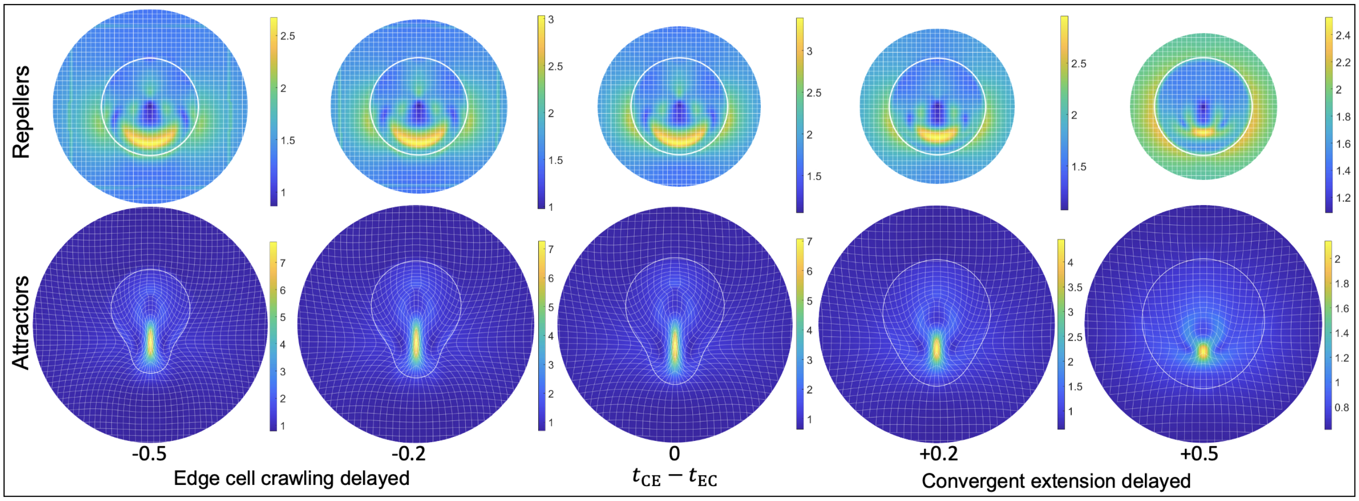

Supplementary Figure S13: **Variable Timing of Epiboly and Posterior Myosin Elevation.** Model repellers (attractors) with the initial (final, deformed) Lagrangian grid and EP boundary. t_{EC} and t_{CE} indicate the time at which edge cell crawling (epiboly) and convergent extension (posterior myosin elevation) start in the model. t_0 is taken to be the smaller of the two. Columns depict output for different values of $t_{\mathrm{CE}} - t_{\mathrm{EC}}$. If $t_{\mathrm{EC}} > t_{\mathrm{CE}}$, posterior myosin is initially elevated but $v_e = 0$ until $t = t_{\mathrm{EC}}$ (left two columns). If $t_{\mathrm{EC}} < t_{\mathrm{CE}}$, EP myosin is initially uniform, but we add posterior myosin (Gaussian function in Table 2) at $t = t_{\mathrm{CE}}$ (right two columns). In the middle column, $t_{\mathrm{CE}} = t_{\mathrm{EC}} = t_0$. Colormaps show the unnormalized ${}_2\lambda$ fields.

References

[1] M. Serra, S. Streichan, M. Chuai, C. J. Weijer, and L. Mahadevan, "Dynamic morphoskeletons in development," *Proceedings of the National Academy of Sciences*, vol. 117, no. 21, pp. 11444–11449, 2020.

[2] S. Mowlavi, M. Serra, E. Maiorino, and L. Mahadevan, "Detecting lagrangian coherent structures from sparse and noisy trajectory data," *Journal of Fluid Mechanics*, vol. 948, p. A4, 2022.

[3] S. S. Barakat and X. Tricoche, "Adaptive refinement of the flow map using sparse samples," *IEEE transactions on visualization and computer graphics*, vol. 19, no. 12, pp. 2753–2762, 2013.

[4] M. Serra and G. Haller, "Objective eulerian coherent structures," *Chaos: An Interdisciplinary Journal of Nonlinear Science*, vol. 26, no. 5, 2016.

[5] P. J. Nolan, M. Serra, and S. D. Ross, "Finite-time lyapunov exponents in the instantaneous limit and material transport," *Nonlinear Dynamics*, vol. 100, no. 4, pp. 3825–3852, 2020.

[6] C. Sinigaglia, F. Braghin, and M. Serra, "Optimal control of short-time attractors in active nematics," *Physical Review Letters*, vol. 132, no. 21, p. 218302, 2024.

[7] M. Serra, P. Sathe, I. Rypina, A. Kirincich, S. D. Ross, P. Lermusiaux, A. Allen, T. Peacock, and G. Haller, "Search and rescue at sea aided by hidden flow structures," *Nature communications*, vol. 11, no. 1, p. 2525, 2020.

[8] N. P. Mitchell, M. F. Lefebvre, V. Jain-Sharma, N. Claussen, M. K. Raich, H. J. Gustafson, A. R. Bausch, and S. J. Streichan, "Morphodynamic atlas for drosophila development," *bioRxiv*, pp. 2022–05, 2022.

[9] R. Asai, S. Sinha, V. N. Prakash, and T. Mikawa, "Bilateral cellular flows display asymmetry prior to left–right organizer formation in amniote gastrulation," *Proceedings of the National Academy of Sciences*, vol. 122, no. 6, p. e2414860122, 2025.

[10] R. Asai, V. N. Prakash, S. Sinha, M. Prakash, and T. Mikawa, "Coupling and uncoupling of midline morphogenesis and cell flow in amniote gastrulation," *Elife*, vol. 12, p. RP89948, 2024.

[11] F. Sadlo and R. Peikert, "Efficient visualization of lagrangian coherent structures by filtered amr ridge extraction," *IEEE transactions on visualization and computer graphics*, vol. 13, no. 6, pp. 1456–1463, 2007.

[12] M. Serra, G. Serrano Nájera, M. Chuai, A. M. Plum, S. Santhosh, V. Spandan, C. J. Weijer, and L. Mahadevan, "A mechanochemical model recapitulates distinct vertebrate gastrulation modes," *Science Advances*, vol. 9, no. 49, p. eadh8152, 2023.

[13] Y. Nakaya, E. W. Sukowati, Y. Wu, and G. Sheng, "Rhoa and microtubule dynamics control cell–basement membrane interaction in emt during gastrulation," *Nature cell biology*, vol. 10, no. 7, pp. 765–775, 2008.

[14] E. Rozbicki, M. Chuai, A. Karjalainen, F. Song, H. Sang, R. Martin, H. Knölker, M. MacDonald, and C. Weijer, "Myosin-II-mediated cell shape changes and cell intercalation contribute to primitive streak formation," *Nat Cell Biol*, vol. 17, no. 4, p. 397, 2015.

[15] M. Kovács, K. Thirumurugan, P. J. Knight, and J. R. Sellers, "Load-dependent mechanism of nonmuscle myosin 2," *Proceedings of the National Academy of Sciences*, vol. 104, no. 24, pp. 9994–9999, 2007.

[16] N. Noll, M. Mani, I. Heemskerk, S. J. Streichan, and B. I. Shraiman, "Active tension network model suggests an exotic mechanical state realized in epithelial tissues," *Nature physics*, vol. 13, no. 12, pp. 1221–1226, 2017.

[17] H. J. Gustafson, N. Claussen, S. De Renzis, and S. J. Streichan, "Patterned mechanical feedback establishes a global myosin gradient," *Nature Communications*, vol. 13, no. 1, p. 7050, 2022.

[18] R. Sknepnek, I. Djafer-Cherif, M. Chuai, C. Weijer, and S. Henkes, "Generating active t1 transitions through mechanochemical feedback," *Elife*, vol. 12, p. e79862, 2023.

[19] M. F. Norstrom, P. A. Smithback, and R. S. Rock, "Unconventional processive mechanics of non-muscle myosin iib," *Journal of Biological Chemistry*, vol. 285, no. 34, pp. 26326–26334, 2010.

[20] C. Veigel, J. E. Molloy, S. Schmitz, and J. Kendrick-Jones, "Load-dependent kinetics of force production by smooth muscle myosin measured with optical tweezers," *Nature cell biology*, vol. 5, no. 11, pp. 980–986, 2003.

[21] V. Ferro, M. Chuai, D. McGloin, and C. J. Weijer, "Measurement of junctional tension in epithelial cells at the onset of primitive streak formation in the chick embryo via non-destructive optical manipulation," *Development*, vol. 147, no. 3, p. dev175109, 2020.

[22] S. Curran, C. Strandkvist, J. Bathmann, M. de Gennes, A. Kabla, G. Salbreux, and B. Baum, "Myosin ii controls junction fluctuations to guide epithelial tissue ordering," *Developmental cell*, vol. 43, no. 4, pp. 480–492, 2017.

[23] R. M. Herrera-Perez, C. Cupo, C. Allan, A. B. Dagle, and K. E. Kasza, "Tissue flows are tuned by actomyosin-dependent mechanics in developing embryos," *PRX Life*, vol. 1, no. 1, p. 013004, 2023.

[24] M. Duda, N. J. Kirkland, N. Khalilgharibi, M. Tozluoglu, A. C. Yuen, N. Carpi, A. Bove, M. Piel, G. Charras, B. Baum, *et al.*, "Polarization of myosin ii refines tissue material properties to buffer mechanical stress," *Developmental cell*, vol. 48, no. 2, pp. 245–260, 2019.

[25] M. Saadaoui, D. Rocancourt, J. Roussel, F. Corson, and J. Gros, "A tensile ring drives tissue flows to shape the gastrulating amniote embryo," *Science*, vol. 367, no. 6476, pp. 453–458, 2020.

[26] O. Voiculescu, F. Bertocchini, L. Wolpert, R. E. Keller, and C. D. Stern, "The amniote primitive streak is defined by epithelial cell intercalation before gastrulation," *Nature*, vol. 449, no. 7165, pp. 1049–1052, 2007.

[27] J. T. Blankenship, S. T. Backovic, J. S. Sanny, O. Weitz, and J. A. Zallen, "Multicellular rosette formation links planar cell polarity to tissue morphogenesis," *Developmental cell*, vol. 11, no. 4, pp. 459–470, 2006.

[28] A. Ioratim-Uba, T. B. Liverpool, and S. Henkes, "Mechano-chemical active feedback generates convergence extension in epithelial tissue," *arXiv preprint arXiv:2303.02109*, 2023.

[29] A. Munjal, J.-M. Philippe, E. Munro, and T. Lecuit, "A self-organized biomechanical network drives shape changes during tissue morphogenesis," *Nature*, vol. 524, no. 7565, pp. 351–355, 2015.

[30] M. Rauzi, U. Krzic, T. E. Saunders, M. Krajnc, P. Ziherl, L. Hufnagel, and M. Leptin, "Embryo-scale tissue mechanics during drosophila gastrulation movements," *Nature communications*, vol. 6, no. 1, pp. 1–12, 2015.

[31] S. J. Streichan, M. F. Lefebvre, N. Noll, E. F. Wieschaus, and B. I. Shraiman, "Global morphogenetic flow is accurately predicted by the spatial distribution of myosin motors," *Elife*, vol. 7, p. e27454, 2018.

[32] A. Bailles, C. Collinet, J.-M. Philippe, P.-F. Lenne, E. Munro, and T. Lecuit, "Genetic induction and mechanochemical propagation of a morphogenetic wave," *Nature*, vol. 572, no. 7770, pp. 467–473, 2019.

[33] L. Giomi, L. Mahadevan, B. Chakraborty, and M. Hagan, "Banding, excitability and chaos in active nematic suspensions," *Nonlinearity*, vol. 25, no. 8, p. 2245, 2012.

[34] R. Fernandez-Gonzalez, S. de Matos Simoes, J.-C. Röper, S. Eaton, and J. A. Zallen, "Myosin ii dynamics are regulated by tension in intercalating cells," *Developmental cell*, vol. 17, no. 5, pp. 736–743, 2009.

[35] F. Brauns, N. H. Claussen, E. F. Wieschaus, and B. I. Shraiman, "The geometric basis of epithelial convergent extension," *eLife*, vol. 13, 2024.

[36] N. H. Claussen, F. Brauns, and B. I. Shraiman, "A geometric-tension-dynamics model of epithelial convergent extension," *Proceedings of the National Academy of Sciences*, vol. 121, no. 40, p. e2321928121, 2024.

[37] T. Lecuit, P.-F. Lenne, and E. Munro, "Force generation, transmission, and integration during cell and tissue morphogenesis," *Annual review of cell and developmental biology*, vol. 27, pp. 157–184, 2011.

[38] P.-A. Pouille and E. Farge, "Hydrodynamic simulation of multicellular embryo invagination," *Physical biology*, vol. 5, no. 1, p. 015005, 2008.

[39] D. New, "The adhesive properties and expansion of the chick blastoderm," *Development*, vol. 7, no. 2, pp. 146–164, 1959.

[40] J. Downie, “The mechanism of chick blastoderm expansion,” *Development*, vol. 35, no. 3, pp. 559–575, 1976.

[41] T. Chung, *Computational fluid dynamics.* Cambridge university press, 2010.

[42] M. Chuai, G. Serrano Nájera, M. Serra, L. Mahadevan, and C. J. Weijer, “Reconstruction of distinct vertebrate gastrulation modes via modulation of key cell behaviors in the chick embryo,” *Science Advances*, vol. 9, no. 1, p. eabn5429, 2023.

[43] M. Williams, C. Burdsal, A. Periasamy, M. Lewandoski, and A. Sutherland, “Mouse primitive streak forms in situ by initiation of epithelial to mesenchymal transition without migration of a cell population,” *Developmental Dynamics*, vol. 241, no. 2, pp. 270–283, 2012.

[44] A. M. Plum and M. Serra, “Morphogen patterning in dynamic tissues,” *bioRxiv*, pp. 2025–01, 2025.

[45] D. Arendt and K. Nübler-Jung, “Rearranging gastrulation in the name of yolk: evolution of gastrulation in yolk-rich amniote eggs,” *Mechanisms of development*, vol. 81, no. 1-2, pp. 3–22, 1999.

[46] D. R. Shook and R. Keller, “Epithelial type, ingression, blastopore architecture and the evolution of chordate mesoderm morphogenesis,” *Journal of Experimental Zoology Part B: Molecular and Developmental Evolution*, vol. 310, no. 1, pp. 85–110, 2008.

[47] M. Takeuchi, M. Takahashi, M. Okabe, and S. Aizawa, “Germ layer patterning in bichir and lamprey; an insight into its evolution in vertebrates,” *Developmental Biology*, vol. 332, no. 1, pp. 90–102, 2009.

[48] J. M. Starck, J. R. Stewart, and D. G. Blackburn, “Phylogeny and evolutionary history of the amniote egg,” *Journal of Morphology*, vol. 282, no. 7, pp. 1080–1122, 2021.

[49] M. Packard and R. Seymour, “Evolution of the amniote egg,” Academic Press, 1997.

[50] M. S. Cooper and V. C. Virta, “Evolution of gastrulation in the ray-finned (actinopterygian) fishes,” *Journal of Experimental Zoology Part B: Molecular and Developmental Evolution*, vol. 308, no. 5, pp. 591–608, 2007.

[51] R. Bellairs, D. Bromham, and C. Wylie, “The influence of the area opaca on the development of the young chick embryo,” *Development*, vol. 17, no. 1, pp. 195–212, 1967.

[52] C. Alev, Y. Wu, Y. Nakaya, and G. Sheng, “Decoupling of amniote gastrulation and streak formation reveals a morphogenetic unity in vertebrate mesoderm induction,” *Development*, vol. 140, no. 13, pp. 2691–2696, 2013.

[53] A. Michaut, A. Chamolly, A. Villedieu, F. Corson, and J. Gros, “A tension-induced morphological transition shapes the avian extra-embryonic territory,” *bioRxiv*, pp. 2024–02, 2024.

[54] D. Kunz, A. Wang, C. U. Chan, R. H. Pritchard, W. Wang, F. Gallo, C. R. Bradshaw, E. Terenzani, K. H. Müller, Y. Y. S. Huang, *et al.*, “Downregulation of extraembryonic tension controls body axis formation in avian embryos.,” 2023.

[55] L. Solnica-Krezel and W. Driever, “Microtubule arrays of the zebrafish yolk cell: organization and function during epiboly,” *Development*, vol. 120, no. 9, pp. 2443–2455, 1994.

[56] C. Camacho-Macorra, N. Tabanera, E. Sánchez-Bustamante, P. Bovolenta, and M. J. Cardozo, “Maternal vgll4a regulates zebrafish epiboly through yap1 activity,” *Frontiers in Cell and Developmental Biology*, vol. 12, 2024.

[57] D. Psychoyos and C. D. Stern, “Restoration of the organizer after radical ablation of hensen’s node and the anterior primitive streak in the chick embryo,” *Development*, vol. 122, no. 10, pp. 3263–3273, 1996.

[58] S. C. Chapman, J. Collignon, G. C. Schoenwolf, and A. Lumsden, “Improved method for chick whole-embryo culture using a filter paper carrier,” *Developmental dynamics: an official publication of the American Association of Anatomists*, vol. 220, no. 3, pp. 284–289, 2001.

[59] G. Serrano Nájera, *Analysis and modulation of cell behaviours driving avian gastrulation.* PhD thesis, School of Life Sciences, University of Dundee, 2021.

[60] A. M. Eskicioglu and P. S. Fisher, “Image quality measures and their performance,” *IEEE Transactions on communications*, vol. 43, no. 12, pp. 2959–2965, 1995.
