# Supplementary material for: Control of tissue flows and embryo geometry in avian gastrulation
Source: Nat Commun. 2025 Jun 4;16:5174. doi: 10.1038/s41467-025-60249-8 (PMC12137940; doi:10.1038/s41467-025-60249-8)
Supplement: Supplementary file 3 — Description of Additional Supplementary Files [file 41467_2025_60249_MOESM3_ESM.pdf]

Description of Additional Supplementary Files

File Name: Supplementary Movie 1
Description: Time evolution associated with Fig. 2.

File Name: Supplementary Movie 2
Description: Time evolution associated with Fig. 3.

File name: Supplementary Movie 3
Description: Time evolution associated with Fig. 4.

File Name: Supplementary Movie 4
Description: Time evolution associated with Fig. 5.

File Name: Supplementary Movie 5
Description: Time evolution of fields in Supplementary Movie 1 for perturbation with no isotropic myosin activity and no epiboly ($v_e = 0$).

File Name: Supplementary Movie 6
Description: Time evolution of fields in Supplementary Movie 1 for perturbation with no isotropic myosin activity.

File Name: Supplementary Movie 7
Description: Time evolution of fields in Supplementary Movie 1 for perturbation with no EE-EP distinction ($m_0(\mathbf{x}_{EE}) = m_>$).

File Name: Supplementary Movie 8
Description: Time evolution of fields in Supplementary Movie 1 for perturbation with no active alignment or passive relaxation ($p_7 = 0$).

File Name: Supplementary Movie 9
Description: Time evolution of fields in Supplementary Movie 1 for perturbation with no anisotropic myosin activity ($s = 0$).

File Name: Supplementary Movie 10
Description: Time evolution of fields in Supplementary Movie 1 for perturbation with higher fluidity ($p_2 = 0.01$).

File Name: Supplementary Movie 11
Description: Time evolution of fields in Supplementary Movie 1 for perturbation with lower fluidity ($p_2 = 0.5$).

File Name: Supplementary Movie 12
Description: Time evolution of fields in Supplementary Movie 1 for perturbation with stronger epiboly ($v_e = 2$).

File Name: Supplementary Movie 13
Description: Time evolution of fields in Supplementary Movie 1 for perturbation with a constant domain boundary velocity ($v_R = 0.4$).

File Name: Supplementary Movie 14
Description: Time evolution of fields in Supplementary Movie 1 for perturbation with delayed edge cell crawling ($t_{CE} - t_{EC} = -0.5$), corresponding to the leftmost column of Fig. **??**.

File Name: Supplementary Movie 15
Description: Time evolution of fields in Supplementary Movie 1 for perturbation with delayed convergent extension ($t_{CE} - t_{EC} = 0.5$), corresponding to the rightmost column of Fig. **??**.

File Name: Supplementary Movie 16
Description: Time evolution of fields in Supplementary Movie 1 for perturbation with no mechanosensitivity ($p_5 = 0$).

File Name: Supplementary Movie 17
Description: Time evolution of fields in Supplementary Movie 1 for perturbation with linear nematic order relaxation.

File Name: Supplementary Movie 18

Description: Time evolution of fields in Supplementary Movie 1 for perturbation with a line initial condition and no anisotropic myosin activity ($s = 0$).

File Name: Supplementary Movie 19
Description: Time evolution of fields in Supplementary Movie 1 for perturbation decreasing p_1 by 50%.

File Name: Supplementary Movie 20
Description: Time evolution of fields in Supplementary Movie 1 for perturbation increasing p_1 by 50%.

File Name: Supplementary Movie 21
Description: Time evolution of fields in Supplementary Movie 1 for perturbation decreasing p_3 by 50%.

File Name: Supplementary Movie 22
Description: Time evolution of fields in Supplementary Movie 1 for perturbation increasing p_3 by 50%.

File Name: Supplementary Movie 23
Description: Time evolution of fields in Supplementary Movie 1 for perturbation decreasing p_4 by 80% and p_5 by 68%.

File Name: Supplementary Movie 24
Description: Time evolution of fields in Supplementary Movie 1 for perturbation increasing p_4 by 900% and p_5 by 900%.

File Name: Supplementary Movie 25
Description: Time evolution of fields in Supplementary Movie 1 for perturbation decreasing p_6 by 50%.

File Name: Supplementary Movie 26
Description: Time evolution of fields in Supplementary Movie 1 for perturbation increasing p_6 by 50%.

File Name: Supplementary Movie 27
Description: Time evolution of fields in Supplementary Movie 1 for perturbation decreasing p_7 by 50%.

File Name: Supplementary Movie 28
Description: Time evolution of fields in Supplementary Movie 1 for perturbation increasing p_7 by 50%.

File Name: Supplementary Movie 29
Description: Time evolution of bright-field microscope images of 8 wild-type (top) and 8 confined (bottom) embryos. Each box containing an embryo is 8.3 mm wide.
